# Systematic approaches to C-lignin engineering in *Medicago truncatula*

**DOI:** 10.1186/s13068-023-02339-7

**Published:** 2023-06-12

**Authors:** Chan Man Ha, Luis Escamilla-Trevino, Chunliu Zhuo, Yunqiao Pu, Nathan Bryant, Arthur J. Ragauskas, Xirong Xiao, Ying Li, Fang Chen, Richard A. Dixon

**Affiliations:** 1 BioDiscovery Institute and Department of Biological Sciences, University of North Texas, 1155 Union Circle #311428, Denton, TX 76203-5017 USA; 2 Center for Bioenergy Innovation (CBI), Oak Ridge National Laboratory, Oak Ridge, TN 37831 USA; 3 Biosciences Division, Oak Ridge National Laboratory, Oak Ridge, TN 37831 USA; 4 Department of Chemical and Biomolecular Engineering, University of Tennessee, Knoxville, TN 37996 USA

**Keywords:** C-lignin, *Medicago truncatula*, Hairy roots, Co-product, Metabolic engineering, Transgenic plants

## Abstract

**Background:**

C-lignin is a homopolymer of caffeyl alcohol present in the seed coats of a variety of plant species including vanilla orchid, various cacti, and the ornamental plant *Cleome hassleriana*. Because of its unique chemical and physical properties, there is considerable interest in engineering C-lignin into the cell walls of bioenergy crops as a high-value co-product of bioprocessing. We have used information from a transcriptomic analysis of developing *C. hassleriana* seed coats to suggest strategies for engineering C-lignin in a heterologous system, using hairy roots of the model legume *Medicago truncatula*.

**Results:**

We systematically tested strategies for C-lignin engineering using a combination of gene overexpression and RNAi-mediated knockdown in the *caffeic acid/5-hydroxy coniferaldehyde 3/5-O-methyltransferase* (*comt*) mutant background, monitoring the outcomes by analysis of lignin composition and profiling of monolignol pathway metabolites. In all cases, C-lignin accumulation required strong down-regulation of caffeoyl CoA 3-*O*-methyltransferase (CCoAOMT) paired with loss of function of COMT. Overexpression of the *Selaginella moellendorffii* ferulate 5-hydroxylase (*SmF5H*) gene in *comt* mutant hairy roots resulted in lines that unexpectedly accumulated high levels of S-lignin.

**Conclusion:**

C-Lignin accumulation of up to 15% of total lignin in lines with the greatest reduction in CCoAOMT expression required the strong down-regulation of both COMT and CCoAOMT, but did not require expression of a heterologous laccase, cinnamyl alcohol dehydrogenase (CAD) or cinnamoyl CoA reductase (CCR) with preference for 3,4-dihydroxy-substituted substrates in *M. truncatula* hairy roots. Cell wall fractionation studies suggested that the engineered C-units are not present in a heteropolymer with the bulk of the G-lignin.

**Supplementary Information:**

The online version contains supplementary material available at 10.1186/s13068-023-02339-7.

## Background

C-Lignin was discovered in 2012 in the seed coats of vanilla orchid and cacti of the genus *Melocactus* [1]. It is a linear polymer of caffeyl alcohol in which the individual units are joined by benzodioxane structures [1]. C-Lignin has since been shown to occur in seed coats of a range of plant species, including other orchids [2], many other species of cacti [3], members of the Euphorbiaceae (e.g., *Jatropha*, *Ricinus, Aleurites*) [4–6], and the ornamental plant *Cleome hassleriana* [4, 7]. The structure of C-lignin makes it an “ideal” lignin for valorization in the bioprocessing of lignocellulosic biomass [8, 9]. This is because the linkages in C-lignin are stable and the polymer does not fragment and then re-polymerize onto cell wall polysaccharides under the types of pretreatment used in the lignocellulosic industry [9]. Furthermore, being a homopolymer, C-lignin can be chemically degraded to one or a few products (primarily catechyl propane [6, 9, 10]), with advantages for subsequent biological funneling to low molecular weight bioproducts or intermediates for synthesis of biomaterials [10]. Finally, the non-degraded polymer itself has favorable properties as a biomaterial, for example for producing carbon fibers [11]. Because of these properties, there is considerable interest in introducing C-lignin into the cell walls of high-volume bioenergy crops such as poplar or switchgrass. However, such efforts are hampered by our lack of understanding of the biosynthesis and in vivo polymerization of C-lignin.

**Fig. 1:**
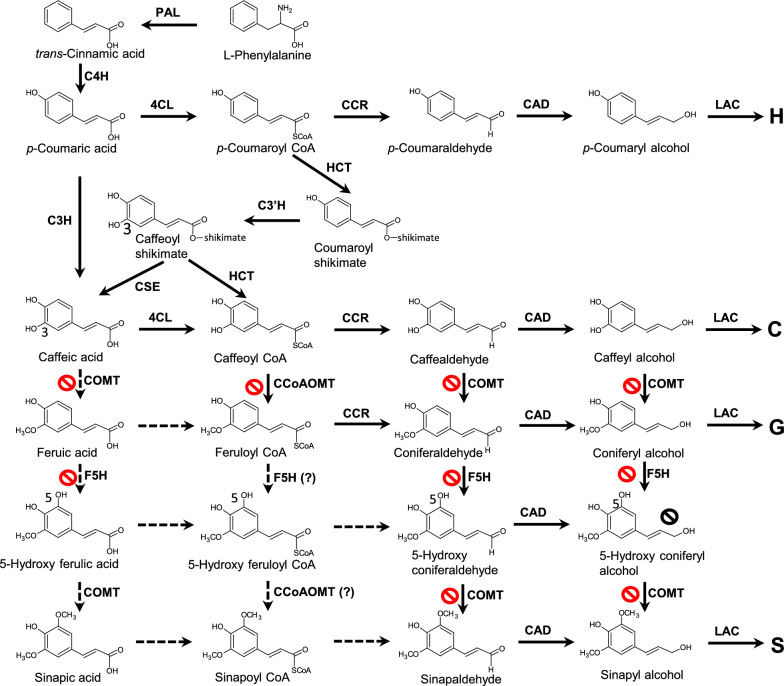
The monolignol biosynthesis pathway, showing all known available routes to H, C, G and S monolignols in dicots. C-lignin is made exclusively of caffeyl alcohol (C) units, biosynthesis of which requires prevention of the *O*-methylation of the 3-OH group by CCoAOMT and/or COMT. The reactions which are blocked during the phase of C-lignin biosynthesis in the Cleome seed coat are shown with a  sign. The 3-OH group in caffeyl alcohol can be introduced by the direct action of C3H on coumarate, or by the combined activities of HCT, C3′H and CSE in the so-called “shikimate shunt”. S lignin is not made in the Cleome seed coat due to lack of expression of F5H. Enzymes are L-phenylalanine ammonia-lyase (PAL), cinnamic acid 4-hydroxylase (C4H), hydroxycinnamoyl CoA: shikimate hydroxycinnamoyl transferase (HCT), coumaroyl shikimate 3′-hydroxylase (C3′H), caffeoyl shikimate esterase (CSE), coumarate 3-hydroxylase (C3H), caffeic acid/5-hydroxyconiferaldehyde 3/5-*O*-methyltransferase (COMT), 4-hydroxycinnamate CoA ligase (4CL), caffeoyl CoA 3-*O*-methyltransferase (CCoAOMT), cinnamoyl CoA reductase (CCR), cinnamyl alcohol dehydrogenase (CAD), ferulic acid/coniferaldehyde 5-hydroxylase (F5H), laccase (LAC)

*Cleome hassleriana* has emerged as a model system for understanding C-lignin biosynthesis. The lignin laid down in the seed coat of this species switches from all guaiacyl (G) lignin to all catechyl (C) lignin at around 12 days after pollination (dap) [4]. This switch is associated with the sudden and rapid transcriptional down-regulation of caffeoyl CoA 3-*O*-methyltransferase (CCoAOMT) and caffeic acid/5-hydroxyconiferyl alcohol 3/5-*O*-methyltransferase (COMT) (Fig. 1) [7]. C-Lignin accumulation is also accompanied by a switch in the complement of cinnamyl alcohol dehydrogenase (CAD, Fig. 1) enzymes in the seed coat, from CAD4, with preference for the G-lignin precursor coniferaldehyde, to CAD5, with preference for the C-lignin precursor caffealdehyde [7]. Finally, polymerization of C-lignin is associated with the appearance, at around 12 dap, of LACCASE 8, a copper oxidase that preferentially oxidizes caffeyl alcohol and which promotes C-lignin formation from exogenously supplied caffeyl alcohol in transgenic *Arabidopsis thaliana* [12]. Caffeyl alcohol is a potent inhibitor of laccase-mediated polymerization of coniferyl alcohol [13], suggesting that C-lignin will be formed preferentially given a suitable supply of caffeyl alcohol in the apoplast.

Here, we build upon the existing knowledge of natural C-lignin biosynthesis to evaluate strategies for C-lignin engineering in non-seed tissues of the model legume *Medicago truncatula*. Lignin biosynthesis has been studied extensively in this species, and in its commercial relative alfalfa (*M. sativa*), primarily in the contexts of understanding systems-wide impacts of lignin modification [14] and engineering reduced lignin alfalfa with improved forage digestibility [15]. *M. truncatula* has an extensive collection of transposon insertion mutants [16], and a rapid system for evaluating gene candidates through expression of transgenes in hairy root cultures [17–19]. Here we use the hairy root system to identify which of the genetic changes associated with C-lignin biosynthesis in *C. hassleriana* is necessary for the accumulation of C-lignin in a heterologous host. Our results indicate that blockage of the classical COMT/CCoAOMT route for monolignol *O*-methylation is sufficient to allow C-lignin biosynthesis. Our studies also revealed that redundant *O*-methylation pathways exist for formation of coniferyl alcohol, and for S-lignin following overexpression of a promiscuous ferulate 5-hydroxylase in the *comt* mutant background. The implications of these results for C-lignin engineering are discussed.

## Materials and methods

### Plant materials and growth conditions

*Tnt1* retrotransposon insertion mutants [16] of *comt*, *ccoaomt*, and *comt ccoaomt* double-mutant plants in *M. truncatula* were as described previously [14]. *M. truncatula* ecotype R108 was used as the wild type for comparison with *Tnt1* insertion mutants. Seed scarification, planting, and plant growth conditions (in a growth chamber set at a 16-h/8-h day/night cycle at a 22 °C [day]/20 °C [night] photoperiod and 70–80% relative humidity) were performed as described [20].

### Plasmid construction and hairy root transformation

The cDNA sequences of *M. truncatula HCT* (Medtr2g105330), *COMT* (Medtr3g092900) and *CCoAOMT* (Medtr4g085590) were synthesized by Gene Universal (https://www.geneuniversal.com) and cloned into pBlueScript II SK(+) to generate RNAi constructs. These were cloned into PCR8/GW/TOPO vector (https://www.thermofisher.com) and finally cloned into pB7GWIWG2 binary vector (HCT-COMT RNAi) or pH7GWIWG2 binary vector (COMT-CCoAOMT RNAi) containing the CaMV 35S promoter by LR recombination reaction (https://gatewayvectors.vib.be). The SmF5H overexpression plasmid (pCC0923) was a kind gift from Dr. Jing-Ke Weng, MIT, USA. For the ChCAD5 overexpression construction, p35S::ChCAD5 (cDNA)::tNOS sequence was synthesized by Gene Universal and cloned into pCC0923 vector using the HiFi DNA assembly protocol (https://www.neb.com/applications/cloning-and-synthetic-biology/dna-assembly-and-cloning/nebuilder-hifi-dna-assembly).

For construction of the ChLAC8 overexpression plasmid (pK7WG2D-ChLAC8), amplified cDNA of ChLAC8 from Cleome stem tissue was first cloned into pENTR/D-TOPO vector (https://www.thermofisher.com) and then cloned into pK7WG2D binary vector containing the CaMV 35S promoter by LR recombination. Then, amplified cDNA of ChLAC15 from Cleome stem tissue was cloned into pK7WG2D-ChLAC8 binary vector using the HiFi DNA assembly protocol to construct the pK7WG2D-ChLAC8/ChLAC15 double overexpression plasmid.

A *GUS* gene was used to construct GUS-pH7GWIWG2 and GUS-pK7WG2D binary vectors as control. The binary vectors described above, singly and in combination, were transformed into *Agrobacterium rhizogenes* strain ARqua1 and transformed colonies were used to inoculate radicles of R108 wild-type and *comt* mutant *M. truncatula* seedlings (NF11911, *Mtcomt-1* allele and NF17882, *Mtcomt-2* allele) to induce hairy root development as described previously [21]. The resulting hairy roots were maintained on B5 medium before sampling. Although we were transforming the *comt* mutants, we included the COMT-RNAi sequence to allow translation to other species for which mutants may not be available.

Primers for cloning and genotyping of transgenic lines are listed in Additional file 1: Table S1.

### Gene transcript analysis

RNAs from plant tissues were isolated using Plant RNA Reagent (Invitrogen). Isolated RNAs were treated with DNase I and then purified by RNeasy MinElute Cleanup Kit (Zymoresearch). Cleaned RNAs were used to perform reverse transcription with SuperScript III reverse transcriptase (Invitrogen). qRT-PCR analysis with Power SYBR Green PCR Master Mix (Life Technologies) was confirmed with three biological replicates using primers described in Additional file 1: Table S1 and performed on an QuantStudio 6 Flex Real-Time PCR system (Life Technologies) according to the manufacturer’s instructions.

### Metabolite extraction and quantification

Lyophilized and ground root powder was weighed (10 mg) and mixed with 1 mL of 60% (v/v) methanol containing ^13^C-benzoic acid as an internal standard. To mix evenly, vortexing was carried out for 30 s, then sonication was applied for 1 h in cold-water. Next, sample tubes were rotated for 1 h at 4 °C and then centrifuged for 15 min at 12,000 rpm. Supernatants were transferred into new tubes and vacuum dried for 3 h. Dried samples were dissolved in 100 μL of 60% (v/v) methanol and subjected to UHPLC–MS/MS analysis at the Bioanalytical Facility of the BioDiscovery Institute, University of North Texas, using an ultra-high performance liquid chromatography tandem mass spectrometry (UHPLC–MS/MS) method as described by Cocuron et al. [22], using an Agilent 1290 Infinity II liquid chromatography system coupled to a hybrid Triple Quadrupole 6500 + triple quadrupole from ABSciex. Compounds were identified and quantified using a mixture of known external standards run at the same time as the biological extracts. Three biological replicates were processed for each tissue.

### Determination of lignin content and composition

Lignin content and monomer composition of freeze-dried hairy root samples were determined using a high-throughput thioacidolysis procedure [23]. Thioacidolysis was also performed on extractive-free samples after additional extraction by the following protocol. The samples were ball milled in a vibrational ball mill for 20 min followed by enzymatic hydrolysis with cellulase from *Trichoderma* sp. in 50 mM acetate buffer (pH 4.8, 37 °C) for 48 h [24]. The solid residue was isolated by centrifugation, washed two times with 6 mM EDTA and two times with deionized water, and freeze-dried for 72 h.

### Testing F5H activity in hairy root microsomes

Isolation of microsomes was carried out as described previously [25], starting with 5 g of either SmF5H overexpressing or control (GUS) hairy roots. C4H and F5H activity assays were conducted combining the methods described by Humphreys et al. [26] and Wang et al. [27] using an NADPH generating system. For C4H assay, 50 µM cinnamic acid was substrate in 100 mM potassium phosphate buffer pH 7.0, whereas 50 µM coniferaldehyde or 50 µM feruloyl CoA were used as substrates for F5H assays; various modifications of incubation time, buffer pH, and amount of microsome preparation were made in a 200 µL total reaction. The reaction mixture with feruloyl CoA was clarified after incubation by centrifugation at 20,000 g for 5 min and subjected to CoA hydrolysis by adding 25 µL of 5 M NaOH and incubating for 20 min at 40 °C; the reaction was then acidified with 175 µL of 1 M HCl. The remaining assays were stopped with 20 µL of glacial acetic acid and clarified by centrifugation at 20,000 g for 5 min. All reactions were extracted twice with 500 µL of ethyl acetate which was reduced to dryness under nitrogen flow, resuspended in 80 µL of methanol and analyzed on an Agilent 1220 HPLC using a reverse-phase C18 column (Spherisorb 5 µm ODS2, Waters, Milford, MA) in a step gradient using 0.1% formic acid in water as solvent A and acetonitrile as solvent B.

### Statistical analysis

Statistical analysis was done by Student’s *t*-test and one-way ANOVA using the Prism program (graphpad.com), *P* < 0.0001.

## Results

### Loss-of-function of OMTs increases C-lignin levels in young seedlings of *M. truncatula*

C-lignin accumulation in the *C. hassleriana* seed coat is associated with a rapid decrease in expression of COMT and CCoAOMT (Fig. 1) [4, 7]. We therefore asked to what extent complete loss of function of these two enzymes would increase the C-lignin content of *M. truncatula* seedlings. We have previously described a series of *M. truncatula* retrotransposon insertion mutants with defects in different steps of the monolignol biosynthesis pathway [14]. This set, in the R108 genetic background, includes individual null mutants in the *COMT* (Medtr3g092900) and *CCoAOMT* (Medtr4g085590) genes, as well as the *comt ccoaomt* double mutant, which cannot survive to maturity [14]. Loss-of-function of *COMT* leads to a massive reduction in S lignin levels in mature plants [14, 28], but 10-day-old *M. truncatula comt* mutant seedlings are still able to make some S-lignin, although at much reduced levels [14].

These previous studies did not measure C-lignin. We therefore analyzed 10-day-old seedlings of wild-type ecotype R108, the *comt*, *ccoaomt*, and c*omt ccoamt* mutants, and mature stem material of R108 for comparison. We first measured selected monolignol pathway enzyme gene transcripts by quantitative real time RT-PCR (qPCR). In comparison to transcript levels in mature stems, transcripts encoding hydroxycinnamoyl CoA: shikimate hydroxycinnamoyl transferase (HCT), CCoAOMT and ferulate/coniferaldehyde 5-hydroxylase (F5H, the entry point into S lignin biosynthesis) [29] were extremely low in all mutants and wild type at 10 days (Additional file 1: Fig. S1A, C, E). In contrast, transcript levels of COMT and CAD5 (the ortholog of the CAD implicated in C-lignin biosynthesis in the Cleome seed coat [7]) in wild-type seedlings were within an order of magnitude of the values in mature stems of wild-type plants (Additional file 1: Fig. S1 B, D). COMT and CCoAOMT transcripts were absent in their individual mutants, reflecting the situation in the Cleome seed coat during C-lignin biosynthesis [7].

**Fig. 2:**
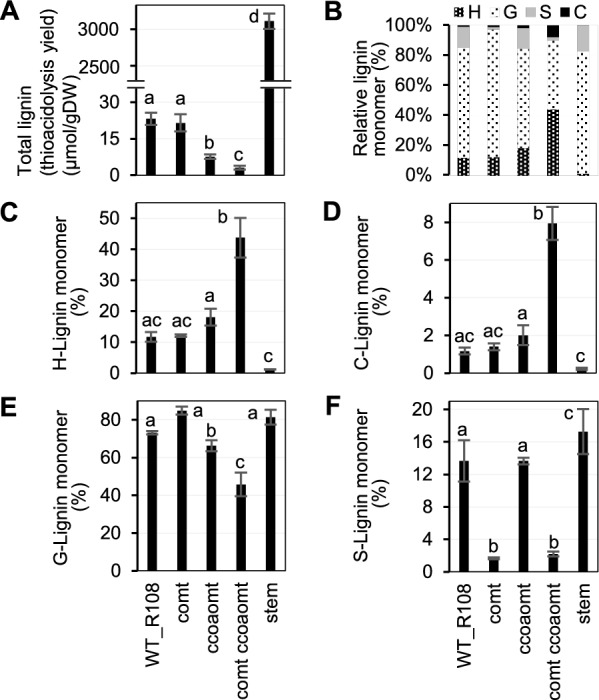
Lignin content and composition in shoots of 10-day-old seedlings of wild-type and OMT mutant *M. truncatula* lines. **A** Total lignin thioacidolysis yield. **B** Overall % lignin monomer compositions. **C** % of H-units. **D** % of C-units. **E** % of G-units. **F** % of S-units. Data are shown as the mean ± sd (for *n* = 3 biologically independent samples); the different letters above the bars represent statistically significant differences determined by one-way analysis of variance (ANOVA; least significant difference (LSD), *P* < 0.0001). Stem, stem tissue from R108 wild-type background

Analysis of monolignol content and composition by thioacidolysis indicated that 10-day-old seedlings of all mutants had very low levels of lignin compared to mature stems, with lowest levels in the *comt coaomt* double mutant (Fig. 2A). The percentage of H-units increased in the mutants in the order *comt*, *ccoaomt* and *comt ccoaomt* (Fig. 2B, C). The percentage of G-units was reduced following loss of function of *CCoAOMT* and, as expected [30, 31], S-units were very low following loss of function of *COMT* (Fig. 2E, F). C-units were present at around 1.8% of total monolignol units in 10-day-old R108 seedlings. However, this proportion was more than fourfold higher in the *comt ccoaomt* double mutant, although the absolute amount of lignin was much reduced (Fig. 2A, D).

**Fig. 3:**
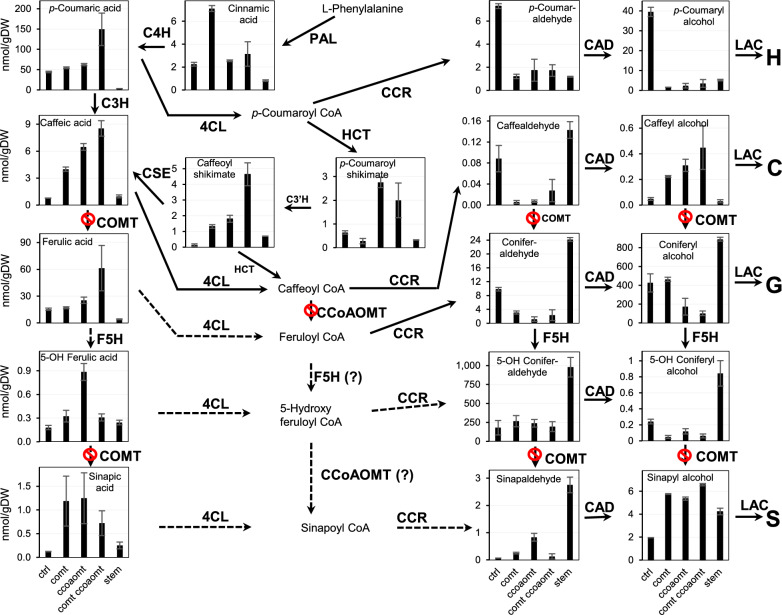
Monolignol pathway metabolite levels in shoots of 10-day-old seedlings of wild-type and OMT mutant *M. truncatula* lines. The figure shows metabolite levels superimposed on a scheme of the monolignol pathways. Metabolite levels were determined by LC–MS/MS transitions pre-determined for individual compounds and quantified by comparison to authentic standards. Structures are shown in Fig. 1. Data are means ± SD derived from three biological replicates. stem, stem tissue from R108 wild-type background

The same tissue samples were then subjected to HPLC–MS/MS analysis to determine the levels of monolignol pathway intermediates (Fig. 3). The structures of the intermediates are shown in Fig. 1. The levels of aldehydes and alcohols (monolignols) did not directly reflect the lignin compositions of the mutants (Fig. 3A-J). Levels of coumaraldehyde and coumaryl alcohol were highest in the wild-type seedlings, but were much lower, closer to the value in mature stems of R108, in all the mutant lines (Fig. 3A, F), even though the mutants had elevated levels of H-units in their lignin (Fig. 2C). In contrast, levels of caffeyl alcohol in the mutants increased in the order *comt*, *ccoaomt* and *comt ccoaom*t (Fig. 3G)*,* consistent with the blocking of the *O*-methylation steps. Nevertheless, the levels of caffeyl alcohol were several orders of magnitude lower than those of coniferyl alcohol, which showed an inverse change in the three mutants compared to caffeyl alcohol (Fig. 3H). After coniferyl alcohol, 5-hydroxyconiferaldehyde, regarded as the preferred substrate for COMT [32] was the most abundant of the pathway intermediates analyzed, but its levels did not change between wild-type and mutant lines (Fig. 3D). Paradoxically, although the other 3,4-dihydroxy-substituted intermediates caffeic acid (Fig. 3O) and caffeoyl shikimate (Fig. 3N) were highest in the *comt ccoaomt* double mutant as would be predicted, so was the mono-methylated intermediate ferulic acid (Fig. 3P), and levels of the dimethylated sinapyl alcohol and sinapic acid were much higher in all three mutants than in wild type (Fig. 3J, R). Together, these results raise the question of the mechanism for monolignol *O*-methylation at the 3 and 5-positions in the absence of functional COMT and CCoAOMT. Potential redundancy in methylation pathways might limit the ability to generate caffeyl alcohol.

### A system for evaluating metabolic engineering strategies for C-lignin biosynthesis

The lack of viability of whole plants lacking function of both COMT and CCoAOMT [14] necessitates an alternative experimental system for rapidly testing combinatorial expression of gene candidates for C-lignin engineering. Hairy roots provide a convenient system for assessing the impact of gene manipulations in plant specialized metabolism [19, 33], and *M. truncatula* hairy roots can be generated quickly and maintained easily [21, 34]. For the present studies, we therefore generated a series of hairy root lines in the *comt* mutant background (using independent *comt-1* and *comt-2* mutant alleles).

### Re-routing of the monolignol pathway by OMT down-regulation coupled with expression of Cleome LACCASE 8

Based on the data in Figs. 2D and 3G, we reasoned that co-down-regulation of COMT and CCoAOMT should increase formation of caffeyl alcohol in *M. truncatula* hairy roots, which, in the presence of a suitable laccase or peroxidase enzyme, may be polymerized to C-lignin. ChLAC8 is specifically expressed in the Cleome seed coat during the phase of C-lignin accumulation and promotes C-lignin accumulation in transgenic Arabidopsis stems fed caffeyl alcohol [12]. We therefore transformed *M. truncatula* hairy roots (*comt* mutant) with a binary vector containing inverted repeat structures for RNA interference of both COMT and CCoAOMT (driven by the constitutive 35S promoter), plus an overexpression cassette for 35S promoter-driven expression of ChLAC8 (Additional file 1: Fig. S2). The dual transformation strategy is described in Methods. The use of two separate plasmids for the overexpression and RNAi constructs resulted in some lines with only the RNAi construct (−/+), some with only the overexpression construct (±), some with both (+/+) and some with neither (−/−). Additional controls included a line with GUS expressed in the *comt* mutant background, and one with GUS in the wild-type R108 background. qPCR analysis revealed baseline COMT transcript levels in all lines, a subset of lines with no CCoAOMT expression, and ChLAC8 expression in all lines transformed with the ChLAC8 construct, although the lines with combined LAC8 overexpression and OMT RNAi had lower LAC8 transcript levels than the lines expressing LAC8 alone (Additional file 1: Fig. S3). The reason for this is not clear.

**Fig. 4:**
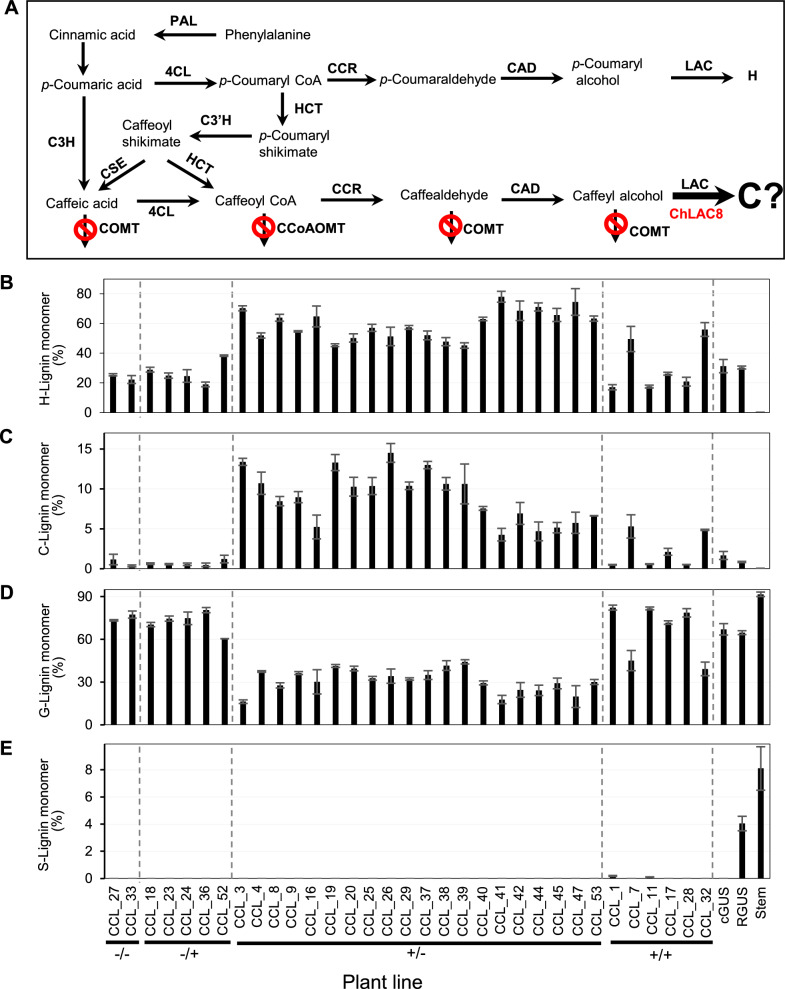
Engineering lignin composition in *M. truncatula* hairy routes by transformation with MtCOMT-MtCCoAOMT RNAi and ChLAC8 overexpression constructs in the *comt* mutant background. **A** Lignin biosynthesis pathway designed for accumulation of C-lignin. The reactions which are blocked during the phase of C-lignin biosynthesis in the Cleome seed coat are shown with a  sign. **B**–**E** Monolignol compositions of transgenic lines determined by thioacidolysis. Data show % of H-lignin monomer (**B**), C-lignin monomer (**C**), G-lignin monomer (**D**) and S-lignin monomer (**E**). cGUS, GUS control in *comt* mutant background; RGUS, GUS control in R108 wild-type background. Stem, mature R108 stem. −/−, no transgenic event; −/+, transgenic roots only harboring ChLAC8 overexpression construct; ±, transgenic roots only harboring MtCOMT-MtCCoAOMT RNAi construct; +/+, transgenic roots harboring both MtCOMT-MtCCoAOMT RNAi and ChLAC8 overexpression constructs. CCL, RNAi construct *for MtCOMT* (C) and *MtCCoAOMT* (C) and overexpression construct for *ChLAC8* (L). Data are means ± SD derived from three biological replicates

Hairy roots expressing COMT and CCoAOMT RNAi constructs appeared a darkish brown color, in contrast to the light-yellow color of GUS empty vector controls in the *comt* mutant background (Additional file 1: Fig.S4). Thioacidolysis revealed essentially no S units in the lignin from all *comt* mutant lines (Fig. 4E), and high C-units (up to 15% of total monolignol units) in all lines in which CCoAOMT was strongly down-regulated (Fig. 4C). There was an inverse relationship between C and G unit levels in these lines (Fig. 4C, D, Additional file 1: Fig. S5), and H unit levels were highest in COMT/CCoAOMT-RNAi lines (Fig. 4B). However, there did not appear to be any relationship between C-lignin accumulation and ChLAC8 expression in lines with low CCoAOMT expression (Fig. 4C, Additional file 1: Fig. S3C). Thus, strong down-regulation of both COMT and CCoAOMT appeared essential for C-lignin accumulation but, unlike the situation in transgenic Arabidopsis [12], ChLAC8 was not required.

**Fig. 5:**
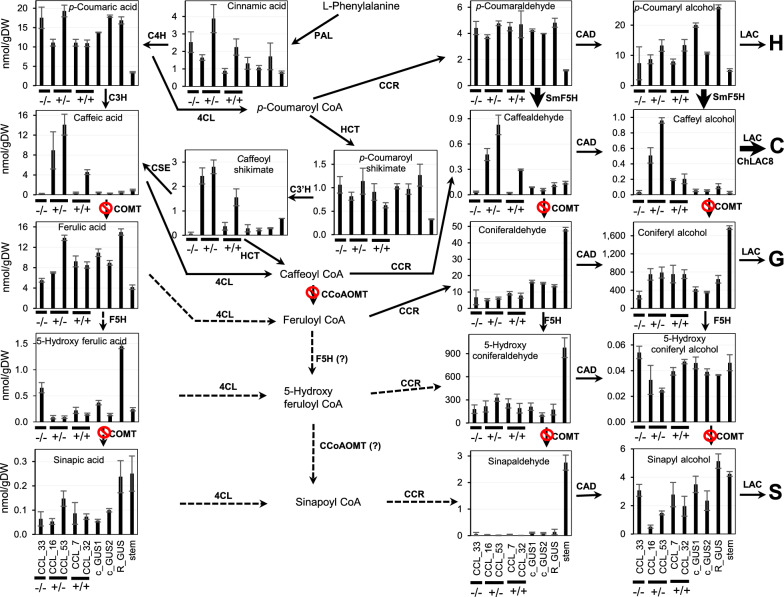
Monolignol pathway metabolite levels in *M. truncatula* MtCOMT-MtCCoAOMT RNAi and ChLAC8 overexpression hairy roots. The engineered lines, in the *comt* mutant background, are a selection of those shown in Fig. 4. The Figure shows metabolite levels superimposed on a scheme of the monolignol pathways. Metabolite levels were determined by LC–MS/MS transitions pre-determined for individual compounds and quantified by comparison to authentic standards. Structures are shown in Fig. 1. CCL, RNAi construct *for MtCOMT* (C) and *MtCCoAOMT* (C) and overexpression construct for *ChLAC8* (L). c_GUS, GUS control in *comt* mutant background; R_GUS, GUS control in R108 wild-type background; stem, mature R108 stem (controls, right-hand 4 bars in all panels). −/−, no transgenic event; −/+, transgenic roots only harboring ChLAC8 overexpression construct; ±, transgenic roots only harboring *MtCOMT-MtCCoAOMT* RNAi construct; +/+, transgenic roots harboring both *MtCOMT-MtCCoAOMT* RNAi and *ChLAC8* overexpression constructs. Data are means ± SD derived from three biological replicates

Figure 5 shows monolignol pathway metabolite data for five selected lignin-modified lines and three controls from the above experiment. As expected, the highest levels of caffeic acid, caffealdehyde, caffeyl alcohol, and caffeoyl shikimate were recorded in hairy roots with loss of function of both COMT and CCoAOMT (Fig. 5B, G, N, O). Levels of sinapyl and 5-hydroxyconiferyl moieties were low. Surprisingly, roots accumulating caffeyl alcohol also contained approximately twofold increased levels of coniferyl alcohol (Fig. 5H) despite the inverse relationship between G and C-units in the lignin.

### Re-routing the monolignol pathway by OMT down-regulation coupled with expression of *Selaginella* F5H

**Fig. 6:**
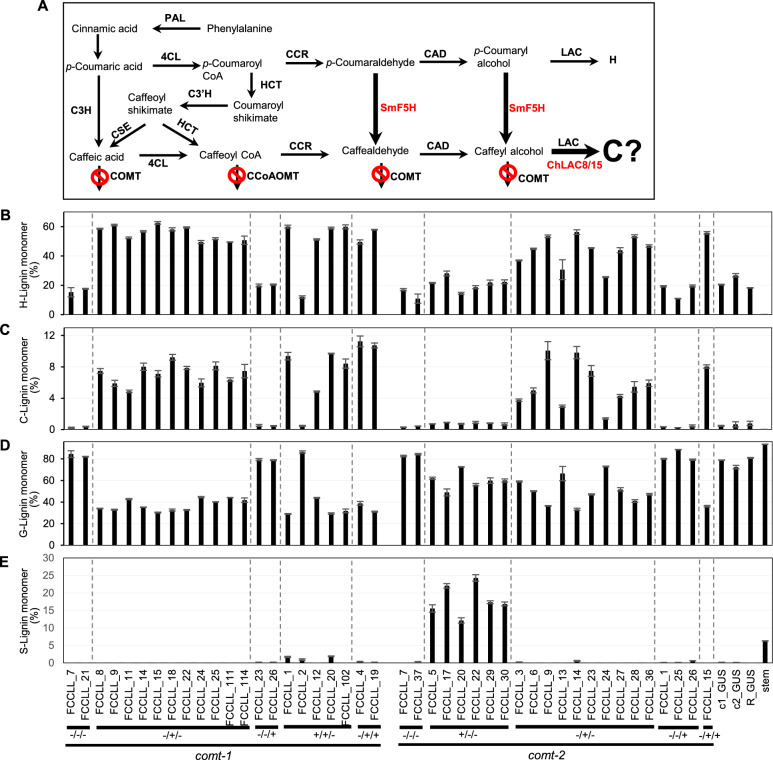
Engineering lignin composition in *M. truncatula* hairy routes by transformation for SmF5H, MtCOMT-MtCCoAOMT RNAi and ChLAC8-ChLAC15 expression in the *comt* mutant background. **A** Lignin biosynthesis pathway designed for accumulation of C-lignin. **B**–**E** Monolignol compositions of transgenic lines determined by thioacidolysis. Data show % of H-lignin monomer (**B**), C-lignin monomer (**C**), G-lignin monomer (**D**) and S-lignin monomer (**E**). c_GUS, GUS control in *comt* mutant background; R_GUS, GUS control in R108 wild-type background. Stem, mature R108 stem. −/−/−, no transgenic event; ±/−, transgenic roots only harboring SmF5H overexpression construct; -/±, transgenic roots only harboring MtCOMT-MtCCoAOMT RNAi construct; −/−/+, transgenic roots harboring only ChLAC8-ChLAC15 *construct; −/*+*/*+*,* transgenic roots harboring both MtCOMT-MtCCoAOMT RNAi and ChLAC8-ChLAC15 overexpression constructs.. FCCLL, overexpression construct for *SmF5H* (F), RNAi construct for MtCOMT-MtCCoAOMT RNAi (CC) and overexpression construct for overexpression for ChLAC8-ChLAC15 (LL). Data are means ± SD derived from three biological replicates

Wild-type *M. truncatula* hairy roots contain high levels (between 25 and 40%) of H-lignin (Fig. 4B) [23]. The *Selaginella moellendorffii* “ferulate 5-hydroxylase” (SmF5H) is a multifunctional enzyme that can uniquely hydroxylate the H-lignin precursors coumaraldehyde and coumaryl alcohol, as well as the S-lignin precursors coniferaldehyde and coniferyl alcohol which are also the substrates for the endogenous *M. truncatula* F5H (MtF5H) [35] (Fig. 1). We reasoned that transformation of *M. truncatula* hairy roots to express SmF5H could provide increased levels of caffealdehyde and caffeyl alcohol (derived from coumaraldehyde and coumaryl alcohol) which, in a COMT down-regulated background, could lead to elevated C-lignin levels (Fig. 6A).

To evaluate the impact of SmF5H expression in a background in which both COMT and CCoAOMT were down-regulated, we performed hairy root transformations with combinations of separate constructs for overexpression of SmF5H (under control of the vascular-specific Arabidopsis cinnamate 4-hydroxylase (C4H) promoter) with RNAi down-regulation of COMT and CCoAOMT (Fig. 6A). We also separately generated lines with a construct designed to introduce the above changes plus expression of both ChLAC15 and ChLAC8 (driven by the strongly root-expressed *Agrobacterium tumefaciens* RolD promoter and the constitutive 35S promoters, respectively) (Additional file 1: Fig. S2); ChLAC15 is also expressed during the phase of C-lignin biosynthesis in the Cleome seed coat [12]. Comparator material included *M. truncatula* hairy root lines transformed with a *GUS* control gene in wild-type and *comt* mutant backgrounds, and wild-type *M. truncatula* mature stems.

To avoid complex and expensive qPCR analysis, we first used a high-throughput thioacidolysis method [23] to phenotype the lines for lignin composition (Fig. 6B–E). Out of the 44 lines generated from transformation with the three constructs, lignin from 28 lines had high levels of C-units (between 3 and 11% of total thioacidolysis units) and, surprisingly, 6 lines had high levels of S-units (between 12 and 24% of total thioacidolysis units (Fig. 6C, E). In all cases, lines that accumulated C-units in their lignin also had elevated levels of H units (Fig. 6B, C), both at the expense of G units (Fig. 6D). Lines making S lignin appeared to do so at the expense of H- and C-lignins.

To understand the basis for these lignin accumulation patterns, we selected 17 lines from those analyzed in Fig. 6 and determined the levels of endogenous *M. truncatula* COMT, CCoAOMT and F5H transcripts, as well as SmF5H and ChLAC8/15 transcripts (Additional file 1: Fig. S6). The results indicate that C-unit accumulation in lignin was associated with strong down-regulation of CCoAOMT transcripts in the *comt* mutant background, but required neither SmF5H nor Cleome LAC8/15 expression. S-Lignin accumulation was associated with expression of SmF5H along with weak to no CCoAOMT down-regulation. Lines expressing both SmF5H-OX and COMT-CCoAOMT-RNAi did not show synergistic effects with respect to accumulation of C- or S-lignin units (Fig. 6C and E).

We next determined the levels of monolignol pathway intermediates in selected lines from the above experiment (Additional file 1: Fig. S7). Caffealdehyde and caffeyl alcohol were present in all lines that accumulated C-units in lignin, although their levels did not directly parallel those of C-lignin. Accumulation of S units in lignin was associated with increased levels of sinapaldehyde, sinapyl alcohol and sinapic acid, but paradoxically also with high levels of 5-hydroxyconiferaldehyde, and 5-hydroxyconiferyl alcohol. Levels of caffeyl alcohol now equaled or exceeded those of coniferyl alcohol in the lines making lignin with a high percentage of C-units (Additional file 1: Fig. S7G, H, J). The intermediates with the highest absolute levels were caffealdehyde in the lines making high C-unit lignin, and 5-hydroxyconiferaldehyde (building up because of the loss of function of COMT) in the lines paradoxically making high S-unit lignin (Additional file 1: Fig. S7D). At the level of the free acids and their esters, coumaric and ferulic acids were much lower in all transgenic lines compared to the GUS controls. Caffeic acid levels were elevated in transgenic lines making C-lignin, and sinapic acid levels were highly elevated in lines making S-lignin. Caffeoyl shikimate levels were highest in lines making lignin rich in C- or S-units.

For simplification, Table 1 presents the metabolite data from selected lines from Additional file 1: Fig. S7 as a heat map showing fold-change compared to the *comt* mutant control. The most striking result is the 40- to 60-fold increase in levels of sinapyl alcohol and sinapic acid in lines overexpressing SmF5H, with their potential direct precursors 5-hydroxyconiferyl alcohol and 5-hydroxyferulic acid /5-hydroxyconiferaldehyde also increasing, although to a lesser extent. Both caffealdehyde and caffeyl alcohol levels were highly increased in lines making C-lignin. Notably, caffeic acid and caffealdehyde levels were among the most strongly down-regulated in SmF5H overexpressing lines.

**Table 1:**
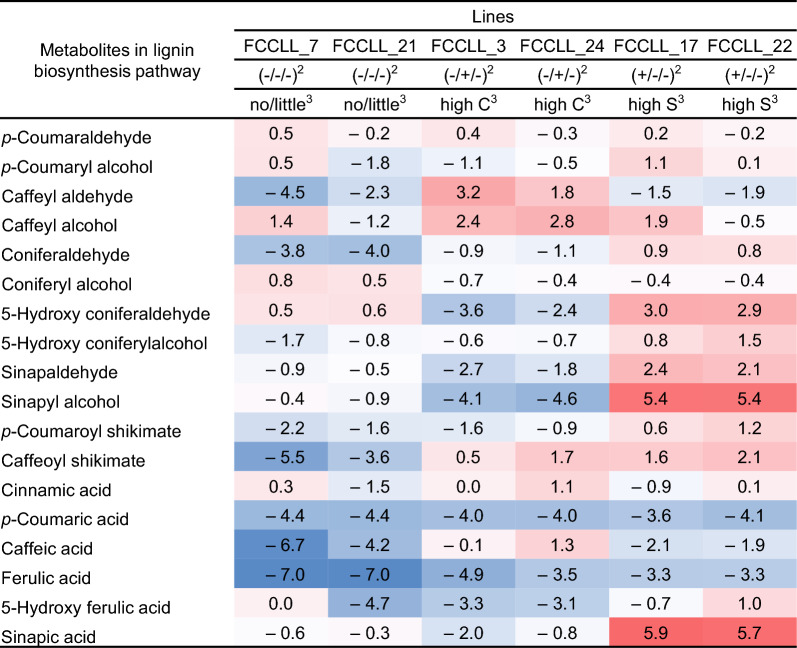
Heatmap of intermediates of the monolignol biosynthesis pathway in engineered transgenic hairy root lines of *M. truncatula* shown in Fig. 6 and Additional file 1: Fig. S7

^1^Values in Table are fold change (Log2 scale) of (nmol/gDW in FCCLL lines)/(nmol/gDW in comt_GUS control lines). Red color indicates relative increase, blue color relative decrease, compared with the control

^2^Genotyping (*SmF5H*-overexpression(F)/*COMT-CCoAOMT* RNAi(CC)/*LAC8-LAC15* overexpression(LL) lines)

^3^Change in lignin content from thioacidolysis analysis. no/little, no or little change in H-,G-,S- and C-lignin; high C, large increase in C-lignin; high S, large increase in S lignin

### HCT down-regulation as a strategy to channel H-monomers to caffeyl alcohol via Selaginella F5H

**Fig. 7:**
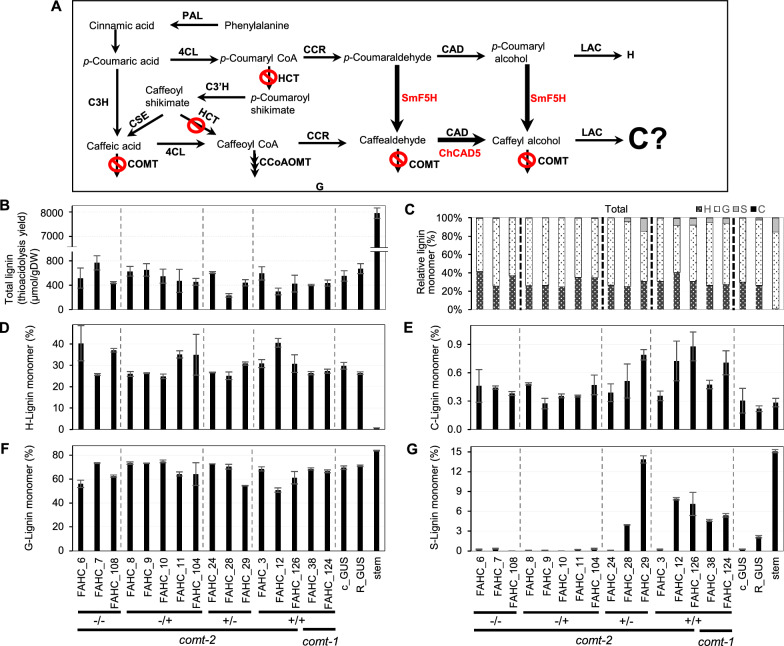
Engineering altered lignin composition in *M. truncatula* hairy roots by down-regulation of COMT and HCT, and overexpression of *Selaginella moellendorffii* F5H (SmF5H) and *Cleome hassleriana* CAD5 (ChCAD5). **A** Scheme showing the predicted pathway. **B**–**G** Monolignol content and composition of selected lines. (**B**) Total lignin thioacidolysis yields; (**C**) overall % lignin monomer compositions; (**D**) % of H-lignin monomers; (**E**) % of C-lignin monomers; (**F**) % of G-lignin monomers; (**G**) % of S-lignin monomers. Hairy root lines designated OR(h) are engineered lines for SmF5H/ChCAD5-OX and HCT/COMT RNAi in the *comt* mutant background. Subsequent genotyping led to selection of plants with no transgenes (−/−), RNAi but not OX transgene (−/+), OX but no RNAi transgene (±), and the presence of both transgenes (+/+). Wild-type R108 and *comt* mutant, both transformed with GUS, served as additional negative controls, and wild-type stems were included for comparison. Transgene constructs are shown in Additional file 1: Fig. S2, and transcript levels of the targeted genes in each line shown in Additional file 1: Fig. S8. FAHC, transformed with overexpression construct for *SmF5H* (F) and *ChCAD5* (A) and RNAi construct for *MtHCT* (H) and *MtCOMT* (C). Data are means ± SD derived from three biological replicates

The lack of a positive impact of SmF5H expression on accumulation of caffeyl alcohol might be explained by preferential conversion of hydroxyphenyl (substrates of SmF5H) to caffeoyl moieties at the CCR/HCT interface (Fig. 1). In this case, levels of H-lignin precursors would be higher if flux into the shikimate shunt was blocked by down-regulation of HCT (Scheme depicted in Fig. 7A). We therefore generated a set of transgenic *M. truncatula* hairy roots with wild-type CCoAOMT expression but in which SmF5H and Cleome CAD5 (for more efficient reduction of caffealdehyde [7]) were over-expressed (under control of the Arabidopsis C4H and 35S promoters, respectively), while HCT and COMT were targeted for RNA interference (under the 35S promoter), all in the *comt* mutant background. Because of the linkage on the same construct, all lines with high SmF5H expression also had high ChCAD5 expression (Additional file 1: Fig. S8D, E). The levels of HCT transcripts were extremely low in all hairy root lines- more than 3 orders of magnitude lower than in mature lignifying stems (Additional file 1: Fig. S8A), and even lower than the level of COMT transcripts determined in the *com*t mutant background (Additional file 1: Fig. S2A, B), consistent with the high levels of H-lignin in the roots. Because of this, it was hard to discern an effect of the HCT-RNAi construct on HCT transcript level. CCoAOMT transcript levels were also very low, reduced in some lines, but variable and seemingly independent of transgene (Additional file 1: Fig. S8C).

Total lignin thioacidolysis yields showed no clear pattern among most of the various transgenic hairy root lines (Fig. 7B). Again, thioacidolysis revealed the presence of S units, to levels much higher than in the wild-type control hairy roots and similar to levels in stems, in lines expressing SmF5H (Fig. 7C, G). However, C-unit levels were low (1% or less of total lignin) in all lines. Levels of G- and H-units did not appear to correlate with genotype (Fig. 7D, F).

Monolignol pathway metabolite levels in the lines described above are shown in Additional file 1: Fig. S9. Levels of sinapic acid, sinapaldehyde and especially sinapyl alcohol (Additional file 1: Fig. S9 R, E, J) were again highly elevated in the lines expressing the SmF5H-ChCAD construct, as were the levels of their precursors 5-hydroxyferulic acid, 5-hydroxyconiferaldehyde and 5-hydroxyconiferyl alcohol (Additional file 1: Fig. S9 Q, D, I).

CCoAOMT is active with 5-hydroxyferuloyl CoA [36], suggesting the possibility of a pathway to the sinapate series via conversion of feruloyl CoA to 5-hydroxyferuloyl CoA. To the best of our knowledge, the activity of Selaginella F5H has not been tested with CoA derivatives. We therefore compared the activities of microsomal extracts from SmF5H-OX and GUS expressing (control) Medicago hairy roots with cinnamate (as a control to show microsomal C4H activity), coniferaldehyde (kinetically the best substrate for SmF5H) [35] and 5-hydroxyferuloyl CoA (assaying for the production of sinapic acid after hydrolysis of the CoA ester). Although the microsomes converted cinnamic acid to coumaric acid in the presence of microsomal extract and an NADPH generating system, no activity was observed for SmF5H-OX or control microsomes with either coniferaldehyde or feruloyl CoA. Likewise, we were unable to show activity of SmF5H with feruloyl CoA after expression in yeast.

### Testing Cleome CCR as a tool for C-lignin engineering

Unusually, the CCR isoform expressed in the Cleome seed coat has a preference for reduction of caffeoyl CoA over coniferyl CoA [7]. We therefore tested whether expression of ChCCR1 in combination with down-regulation of MtCCoAOMT, the enzyme competing for caffeoyl CoA, could enhance C-lignin production in hairy roots of the *M. truncatula comt* mutant, using the constructs shown in Additional file 1: Fig. S2. Over 80 independent hairy root lines were generated, but none showed elevated levels of C-lignin.

### Differential extractability of lignins in engineered hairy roots

The C-monomers accumulating in the lignin of engineered *M. truncatula* hairy roots could be present in a homopolymer as in the native C-lignin of the Cleome or Vanilla seed coat [1, 4], or as a component of a heteropolymer with G, H and S units. To distinguish between these possibilities, we attempted to submit extractive-free hairy root samples to whole cell NMR analysis. To this end, we grew each line of hairy roots in five tissue culture dishes and harvested them after 6 weeks growth (approx. biomass per dish 0.6 g dry wt). Samples of extractive-free freeze-dried hairy roots were selected from three lines depicted in Fig. 4; R(C)L-4 and R(C)L-16 (high in C-units) and R(C)L-20 (high in S-units), along with two GUS-controls. Surprisingly, the C-unit signal was too low to accurately quantify monomer units by NMR. Because this appeared to contradict the initial thioacidolysis data for the samples in Fig. 4, we re-analyzed the samples by thioacidolysis after the cellulase digestion and EDTA extraction used to prepare the materials for NMR. The total lignin (sum of monomer thioacidolysis yields) in the fractions from the GUS control lines was not reduced after enzymatic treatment and EDTA extraction, whereas decreases were seen in the fractions from one of the high C-unit lines and the high S-unit line (Additional file 1: Fig. S10 A). More striking was the large reduction in C-units (in total or as a % of total thioacidolysis units) following enzymatic treatment and EDTA extraction in the two lines engineered to contain C-lignin (Additional file 1: Fig. S10 C, G) in contrast to the lack of effect of this treatment on the % of G-units (Additional file 1: Fig. S10 H). The total and proportion of S units were also decreased by enzyme/EDTA treatment (Additional file 1: Fig. S10E, I), but those of H units were not (Additional file 1: Fig. S10 B, F). Taken together, these data suggest that the C-units in the engineered C-lignin are not incorporated into a G-C co-polymer.

## Discussion

### OMT down-regulation can be sufficient for the accumulation of C-lignin in engineered plants

The present data indicate that simultaneous down-regulation of COMT and CCoAOMT, as occurs at the onset of C-lignin accumulation in *C. hassleriana* [4, 7], is sufficient to result in accumulation of C-units in lignin in seedlings or hairy roots of *M. truncatula*, at percentages around half of that seen in the seed coat of *C. hassleriana* [4]. The additional approaches we attempted to re-direct or enhance the pathway, as discussed below, did not lead to increased C-lignin biosynthesis. In the hairy roots, co-expression of Cleome cell wall laccases associated with C-lignin accumulation did not lead to further polymerization of C-units, suggesting that *M. truncatula* possesses the necessary enzyme(s) for C-lignin polymerization. This is consistent with the small but significant presence of C-lignin in young wild-type *M. truncatula* seedlings. C-lignin has only previously been recorded to occur naturally in seed coats [1, 2, 4–7]. In contrast to *M. truncatula*, Arabidopsis appears to be unable to naturally polymerize caffeyl alcohol to C-lignin, based on the requirement for expression of Cleome LACCASE 8 for generation of C-lignin from exogenously applied caffeyl alcohol in cut stems of this species [12].

### Re-directing flux to C-lignin

Because of its demonstrated activities with 4-coumaryl aldehyde and alcohol [35], we reasoned that expression of SmF5H might help redirect flux from the H monolignol pathway, which is particularly prevalent in *M. truncatula* hairy roots, to C-monolignol precursors. However, expression of SmF5H alone did not result in C-lignin accumulation; rather, up to 24% S-units were incorporated into the lignin, a paradoxical result as the experiments were performed in the *comt* mutant background in which S lignin formation is blocked.

Loss-of-function of COMT accompanied by overexpression of angiosperm F5H results in accumulation of monolignol precursors with 3-methoxy, 4,5-dihydroxy (i.e., 5-hydroxyguaiacyl) substitution, including 5-hydroxyconiferyl alcohol, which can be incorporated into lignin at high levels in these plants [37, 38]. In the present hairy root lines, elevated accumulation of 5-hydroxyconiferaldehyde, the preferred substrate for COMT [26, 39] was as expected for overexpression of an F5H in the absence of COMT. However, 5-hydroxyconiferaldehyde must be further methylated to generate the 3-methoxy, 4,5-dihydroxy moiety of S-lignin, and sinapyl alcohol and sinapic acid accumulated in the SmF5H-OX/*comt* mutant background.

If the pools of H unit precursors were insufficient to support conversion by SmF5H, blocking HCT expression would be expected to direct flux into the H monolignol pathway [40]. Hairy root lines with HCT-RNAi in the absence of SmF5H expression exhibited between approximately 5- and 20-fold increases in 4-coumaryl aldehyde/alcohol, indicating that HCT repression was indeed occurring in spite of the difficulty in showing this by transcript measurement; however, this did not result in increased H-lignin levels, and neither did combining HCT-RNAi with SmF5H-OX.

Overexpression of Cleome CAD5 was included in a construct linked to SmF5H expression because expression of CAD5, which has a preference for caffeyl alcohol, has been shown to correlate with C-lignin accumulation in the Cleome seed coat [7]. However, metabolite and lignin analyses failed to reveal any impact of ChCAD5 expression, particularly as regards C-lignin accumulation, in the hairy roots. A similar conclusion can be made for the lack of efficacy of Cleome CCR1, with preference for caffeoyl CoA, in enhancing C-lignin formation in *M. truncatula*. This is possibly because of the expression in roots of the endogenous Medicago CCR2 enzyme, which, like ChCCR1, exhibits a preference for caffeoyl CoA [41].

Because of the time-consuming nature of plant transformation, even when using a hairy root system, we employed the strategy of co-transformation with up to 3 constructs to introduce the selected genetic changes. Roots were either first genotyped for integration of the various genetic elements, or else phenotyped first to determine the presence or absence of C- or S-lignin, and then positive lines selected for genotyping. It is important to note that, in spite of multiple attempts, we were never able to obtain hairy root lines with both COMT-CCoAOMT RNAi and SmF5H-overexpression. Perhaps additional efforts would help attain this goal. Alternatively, it is possible that, just as loss of function of both COMT and CCoAOMT is ultimately lethal in *A. thaliana* and *M. truncatula* seedlings [14, 42], the same is true as regards initiation of hairy root cultures with additional overexpression of SmF5H.

Although SmF5H overexpression resulted in striking changes in lignin composition and metabolite pools, its combination with HCT-RNAi did not result in the conversion of p-coumaryl to caffeyl moieties. Clearly, the presence of a functional CCoAOMT does not allow redirection of the pathway away from formation of G-units.

### Relation between monolignol pool size and lignin composition

We analyzed monolignol pool sizes based on the assumption that they might be reflective of lignin composition. In *M. truncatula* seedlings, loss of function of either COMT or CCoAOMT resulted in an increase in the caffeyl alcohol pool, but only co-down-regulation of COMT and CCoAOMT resulted in C-lignin accumulation. However, the absolute levels of caffeyl alcohol in the *comt ccoaomt* double mutant were an order of magnitude lower than the levels of sinapyl alcohol, and more than two orders of magnitude lower than the levels of coniferyl alcohol. Because the *comt ccoaomt* double mutant accumulates no S lignin but still makes sinapyl alcohol, it is clear that monolignol pool sizes need not reflect the proportion of the particular monolignol in the lignin polymer. This has recently been observed in other studies where it has been shown that coniferyl alcohol may accumulate due to the inhibition of its polymerization by caffeyl alcohol [13], and monolignol accumulation might be uncoupled from lignin formation if monolignols serve additional defensive functions. The specificity of lignin polymerizing enzymes likely plays a role in determining lignin composition [13, 43], particularly if the transport of monolignols to the apoplast is primarily via passive diffusion [13, 43–45].

### Alternative pathways for monolignol *O*-methylation

All transgenic lines in the present work were in the *comt* mutant background, which does not accumulate S-lignin. Because loss of function or strong down-regulation of CCoAOMT results in accumulation of C-lignin, and overexpression of SmF5H in the absence of CCoAOMT down-regulation in formation of S lignin and accumulation of non-methylated 5-hydroxyguaiacyl intermediates, it seems likely that the *O*-methylation of 5-hydroxyguaiacyl intermediates is being catalyzed by CCoAOMT, at the level of 5-hydroxyferuloyl CoA, a known substrate for the enzyme [36]. This leads to the expectation that SmF5H will be active with feruloyl CoA, but this has, to the best of our knowledge, not yet been reported. Despite many attempts, we were unable to demonstrate microsomal feruloyl CoA or coniferaldehyde 5-hydroxylase activity in extracts from S-lignin accumulating SmF5H-OX lines, although coniferaldehyde is the best substrate for the recombinant enzyme expressed in yeast [35]. The yeast-expressed enzyme has been reported to be inactive with caffeoyl shikimate [35], so it is unlikely that it exhibits activity with the bulkier CoA ester. Other papers have reported inability to demonstrate F5H activity in plant extracts [25, 46], and, to the best of our knowledge, only a single study has provided kinetic data for other than the recombinant enzyme [27]. Formation of sinapyl CoA via CCoAOMT would account for the increased levels of sinapaldehyde (via CCR), sinapic acid (via aldehyde dehydrogenase acting on sinapaldehyde) [47] and sinapyl alcohol (via CAD). 5-Hydroxyferulic acid, 5-hydroxyconiferaldehyde and 5-hydroxyconiferyl alcohol all accumulate because of the loss of function of COMT. It is also clear, however, that *comt* mutant seedlings accumulate small amounts of S-lignin [this work, 14], and seed coats of *C. hassleriana* continue to synthesize coniferyl alcohol after both COMT and CCoAOMT have been switched off [13], suggesting the operation of additional OMT enzymes that remain to be identified. The fact that the pool size of coniferyl alcohol remained much higher than that of caffeyl alcohol in the present *comt ccoaomt* double mutants further supports additional redundancy for monolignol *O*-methylation. This situation reflects redundancy in CAD enzymes shown by the accumulation of H-lignin in Arabidopsis mutants that lack functional copies of all CAD enzymes that have been associated with lignin biosynthesis to date [48].

### The nature of engineered C-rich lignin in *M. truncatula*

The C-lignin in the Cleome seed coat is a homopolymer, laid down after a period of G-lignin synthesis and deposition, and no evidence could be found for the presence of any C-G-heteropolymer [4]. In contrast, C-enhanced lignin in CCoAOMT down-regulated pine cultures is a heteropolymer comprising predominantly G and H units with up to 5.6% of benzodioxane-linked units ascribed to the incorporation of caffeyl alcohol [49]. The material containing C-units in the engineered *M. truncatula* hairy roots is not released by methanol/water extraction and is not therefore of low molecular weight such as the dimeric C-lignans found in the Cleome seed coat [13]. It is, however, extracted by aqueous EDTA after hydrolysis of cell walls with *Trichoderma* cellulase, whereas the G-lignin is not, indicating that the C-units are not part of a G-C co-polymer but likely a separate homopolymeric C-lignin fraction. This fraction is readily removed from cellulose-depleted cell wall material by aqueous extraction. More work is needed to compare the structural features of engineered C-lignins with those of the polymer from natural sources.

## Conclusions

We have demonstrated the engineering of C-lignin in *M. truncatula* hairy roots to a percentage, relative to G-lignin, comparable that of natural sources [4]. This requires only the strong down-regulation of both COMT and CCoAOMT. However, the total overall lignin yield is strongly depressed, and similar genetic manipulation in whole plants results in serious growth defects [14, 42]. The successful development of C-lignin as a value-added product of biorefining will require understanding the basis for the growth defects and incorporating genetic mechanisms for their suppression (reviewed in [50]). It is also likely to require tissue-specific OMT down-regulation. Furthermore, the size of the engineered caffeyl alcohol pool in the hairy roots is disproportionately small compared to the situation in the Cleome seed coat [13], and it is possible that this reflects conversion of caffeyl to coniferyl alcohol in the absence of functional COMT. The accumulation of high levels of S-lignin in *comt* mutant plants expressing SmF5H is also suggestive of OMT redundancy. It will therefore be interesting to further examine the large number of COMT-like proteins encoded in the *M. truncatula* genome [51] to determine which may share redundant roles in monolignol *O*-methylation and could be targets for down-regulation to allow for more efficient C-unit biosynthesis.

## Supplementary Information

**Additional file 1: Figure S1.** Monolignol pathway enzyme transcript levels in 10-day-old seedlings *of M. truncatula*. **Figure S2.** Plasmid constructs used in this study. **Figure S3.** Target gene transcript levels in *M. truncatula* hairy roots engineered for MtCOMT-MtCCoAOMT RNAi and ChLAC8 overexpression in the *comt* mutant background. **Figure S4.** Visible phenotypes of control and C-lignin producing *M. truncatula* hairy roots. **Figure S5.** Scatter plot showing linear regression analysis for the relationship between G-lignin monomer and C-lignin monomer content in *M. truncatula* hairy roots making C-rich lignin. **Figure S6.** Monolignol pathway enzyme transcript levels in *M. truncatula* hairy roots engineered for expression of SmF5H-OX*,* MtCOMT-MtCCoAOMT RNAi and ChLAC8-ChLAC15-OX constructs in the *comt* mutant background. **Figure S7.** Levels of monolignol pathway intermediates in *M. truncatula* hairy roots engineered for SmF5H overexpression, MtCOMT-MtCCoAOMT RNAi and ChLAC8-ChLAC15 overexpression in the *comt* mutant background. **Figure S8.** Monolignol pathway enzyme transcript levels in *M. truncatula* hairy roots engineered for expression of SmF5H-ChCAD5 OX + HCT-COMT RNAi in the *comt* mutant background. **Figure S9.** Monolignol pathway metabolite levels in selected *M. truncatula* SmF5H-ChCAD5 overexpression / MtHCT-MtCOMT RNAi hairy roots. **Figure S10.** Differential extractability of C- and G-lignins from *M. truncatula* hairy roots as determined by thioacidolysis. **Table S1.** Primers used in the present work.

## Data Availability

The data sets supporting the conclusions of this article are included in this article and its Additional file 1.
