# Supplementary material for: Systematic approaches to C-lignin engineering in Medicago truncatula
Source: Biotechnol Biofuels Bioprod. 2023 Jun 12;16:100. doi: 10.1186/s13068-023-02339-7 (PMC10262568; doi:10.1186/s13068-023-02339-7)
Supplement: Supplementary file 1 — Additional file 1: Figure S1. Monolignol pathway enzyme transcript levels in 10-day-old seedlings of M. truncatula. Figure S2. Plasmid constructs used in this study. Figure S3. Target gene transcript levels in M. truncatula hairy roots engineered for MtCOMT-MtCCoAOMT RNAi and ChLAC8 overexpression in the comt mutant background. Figure S4. Visible phenotypes of control and C-lignin producing M. truncatula hairy roots. Figure S5. Scatter plot showing linear regression analysis for the relationship between G-lignin monomer and C-lignin monomer content in M. truncatula hairy roots making C-rich lignin. Figure S6. Monolignol pathway enzyme transcript levels in M. truncatula hairy roots engineered for expression of SmF5H-OX, MtCOMT-MtCCoAOMT RNAi and ChLAC8-ChLAC15-OX constructs in the comt mutant background. Figure S7. Levels of monolignol pathway intermediates in M. truncatula hairy roots engineered for SmF5H overexpression, MtCOMT-MtCCoAOMT RNAi and ChLAC8-ChLAC15 overexpression in the comt mutant background. Figure S8. Monolignol pathway enzyme transcript levels in M. truncatula hairy roots engineered for expression of SmF5H-ChCAD5 OX + HCT-COMT RNAi in the comt mutant background. Figure S9. Monolignol pathway metabolite levels in selected M. truncatula SmF5H-ChCAD5 overexpression / MtHCT-MtCOMT RNAi hairy roots. Figure S10. Differential extractability of C- and G-lignins from M. truncatula hairy roots as determined by thioacidolysis. Table S1. Primers used in the present work. [file 13068_2023_2339_MOESM1_ESM.pdf]

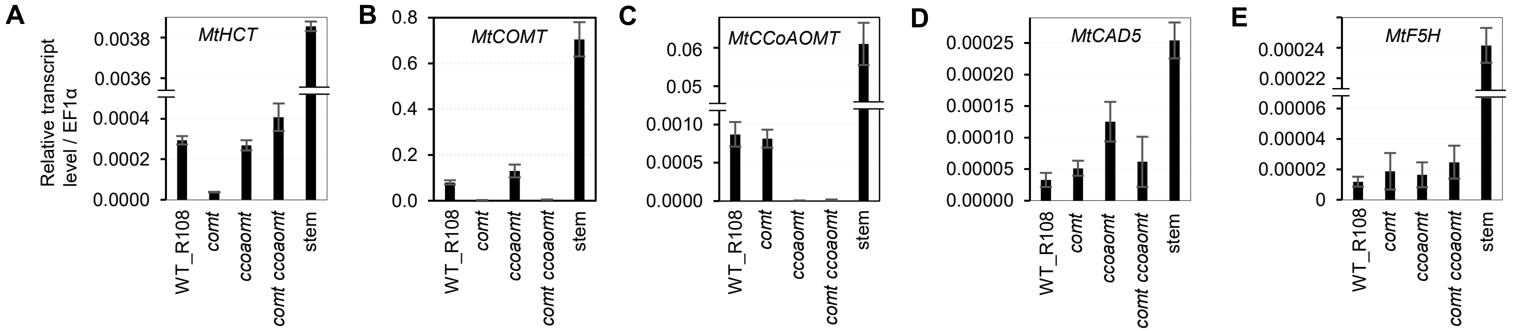

Supplemental Fig. S1. Monolignol pathway enzyme transcript levels in 10-day-old seedlings *of M. truncatula.* Transcript levels were determined by RT-qPCR analysis for *MtHCT* (**A**), *MtCOMT* (**B**), *MtCCoAOMT* (**C**), *MtCAD5* (**D**) and *MtF5H* (**E**) transcripts. Stem, stem tissue of mature wild-type R108 plant. Data are means ± SD derived from three biological replicates.

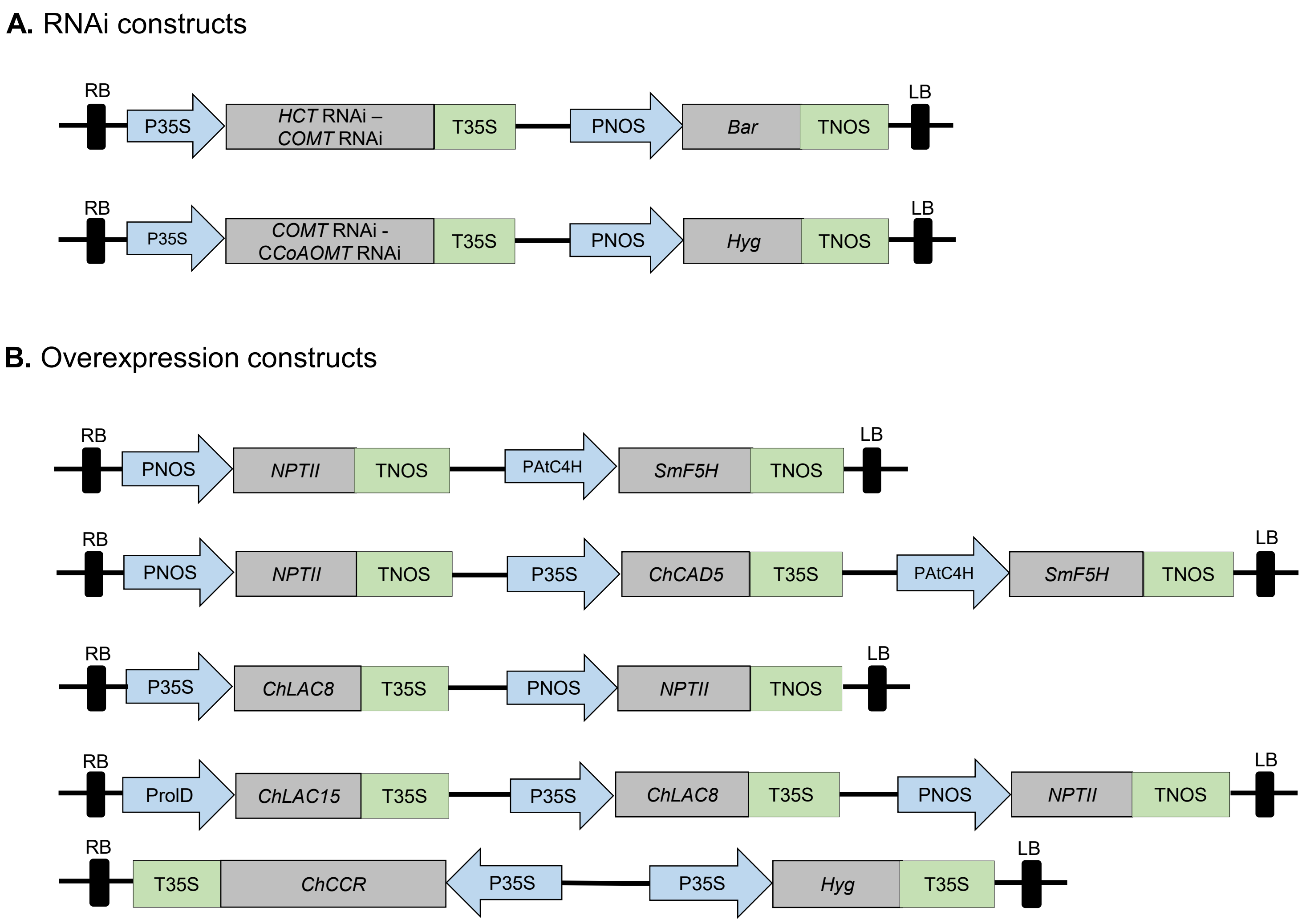

Supplemental Fig. S2. Plasmid constructs used in this study. Diagram of plasmid constructs for RNAi expression cassette (**A**) and overexpression cassette (**B**). Bar, Basta resistance (containing phosphinothricin) gene; Hyg, Hygromycin resistance gene; pAtC4H, promoter of cinnamate 4-hydroxylase gene from *Arabidopsis thaliana*; ProlD, Agrobacterium tumefaciens RolD promoter; NPTII, kanamycin resistance gene.

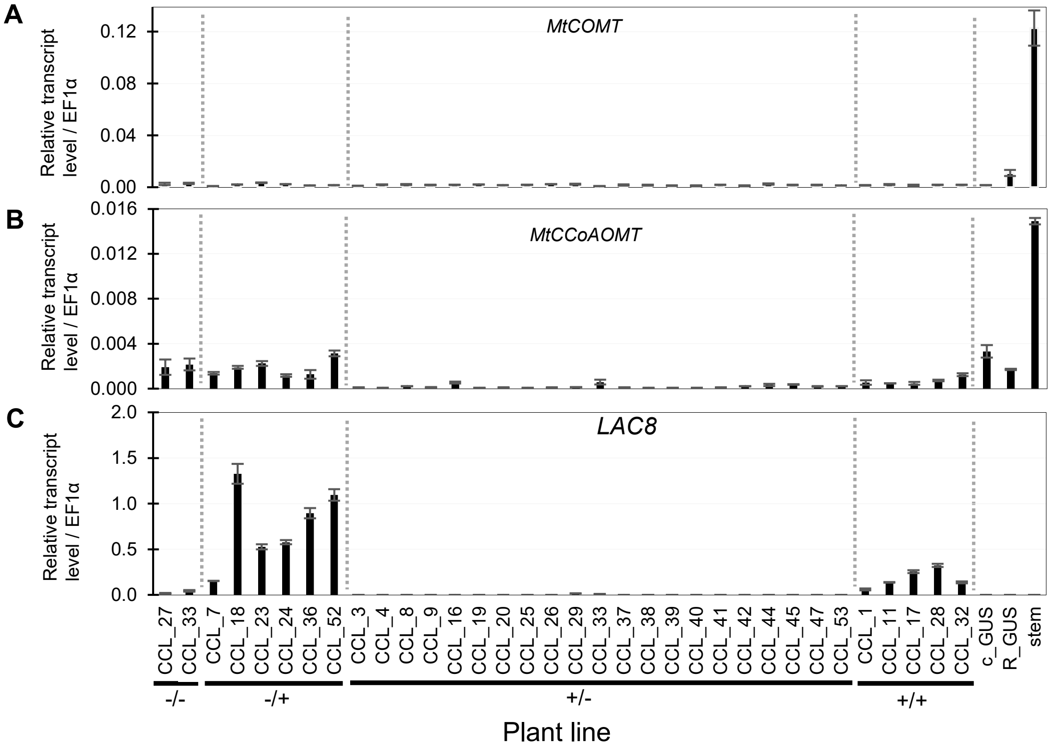

Supplemental Fig. S3. Target gene transcript levels in *M. truncatula* hairy roots engineered for *ChLAC8* overexpression and *MtCOMT-MtCCoAOMT* RNAi in the *comt* mutant background. Transcript levels were determined by qPCR analysis for *MtCOMT* (**A**), *MtCCoAOMT* (**B**), and *ChLAC8* (**C**) transcripts. c_GUS, GUS control in *comt* mutant; R_GUS, GUS control in R108 wild-type background; stem, stem tissue from R108 wild type. CCL, RNAi construct for *MtCOMT* (C) and *MtCCoAOMT* (C) and overexpression construct for *ChLAC8* (L). -/-, no transgenic event; -/+, transgenic roots only harboring *ChLAC8* overexpression construct; +/-, transgenic roots only harboring *MtCOMT-MtCCoAOMT* RNAi construct; +/+, transgenic roots harboring both *MtCOMT-MtCCoAOMT* RNAi and *ChLAC8* overexpression constructs. Data are means ± SD derived from three biological replicates.

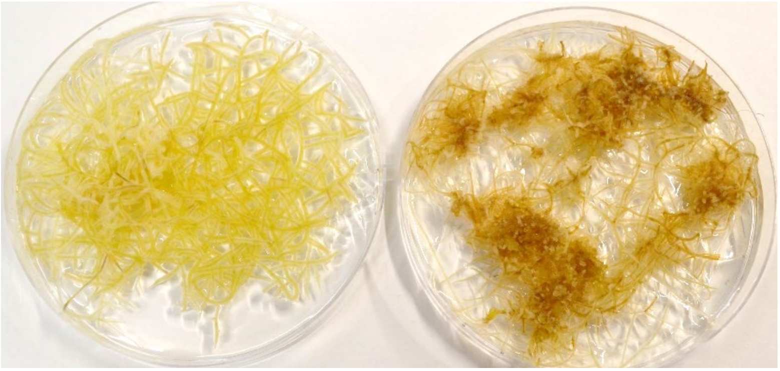

Supplemental Fig. S4. Visible phenotypes of control and C-lignin producing *M. truncatula* hairy roots. Ctrl, control line with GUS vector in *comt* mutant background; RNAi, transgenic line with *MtCOMT-MtCCoAOMT* RNAi in the *comt* mutant background.

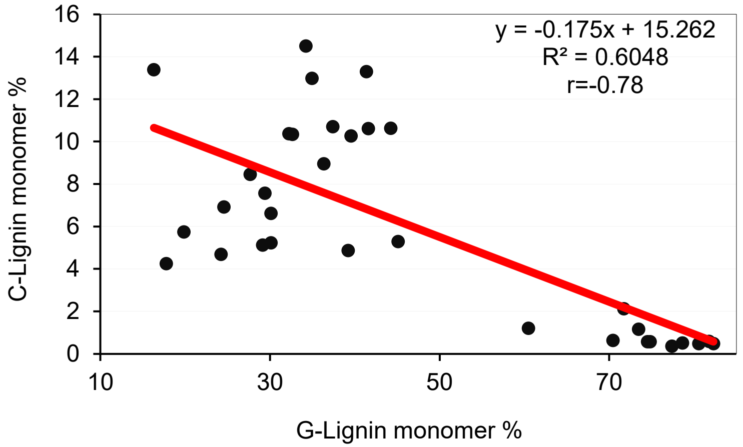

Supplemental Fig. S5. Scatter-plot showing linear regression analysis for the relationship between G -lignin monomer and C-lignin monomer content in *M. truncatula* hairy roots making C-rich lignin. This figure derives from Figs 4C and 4D for *MtCOMT-MtCCoAOMT* RNAi and *ChLAC8* overexpression transgenic lines in the *comt* mutant background, with lignin composition determined by thioacidolysis. r = Pearsons's correlation coefficient.

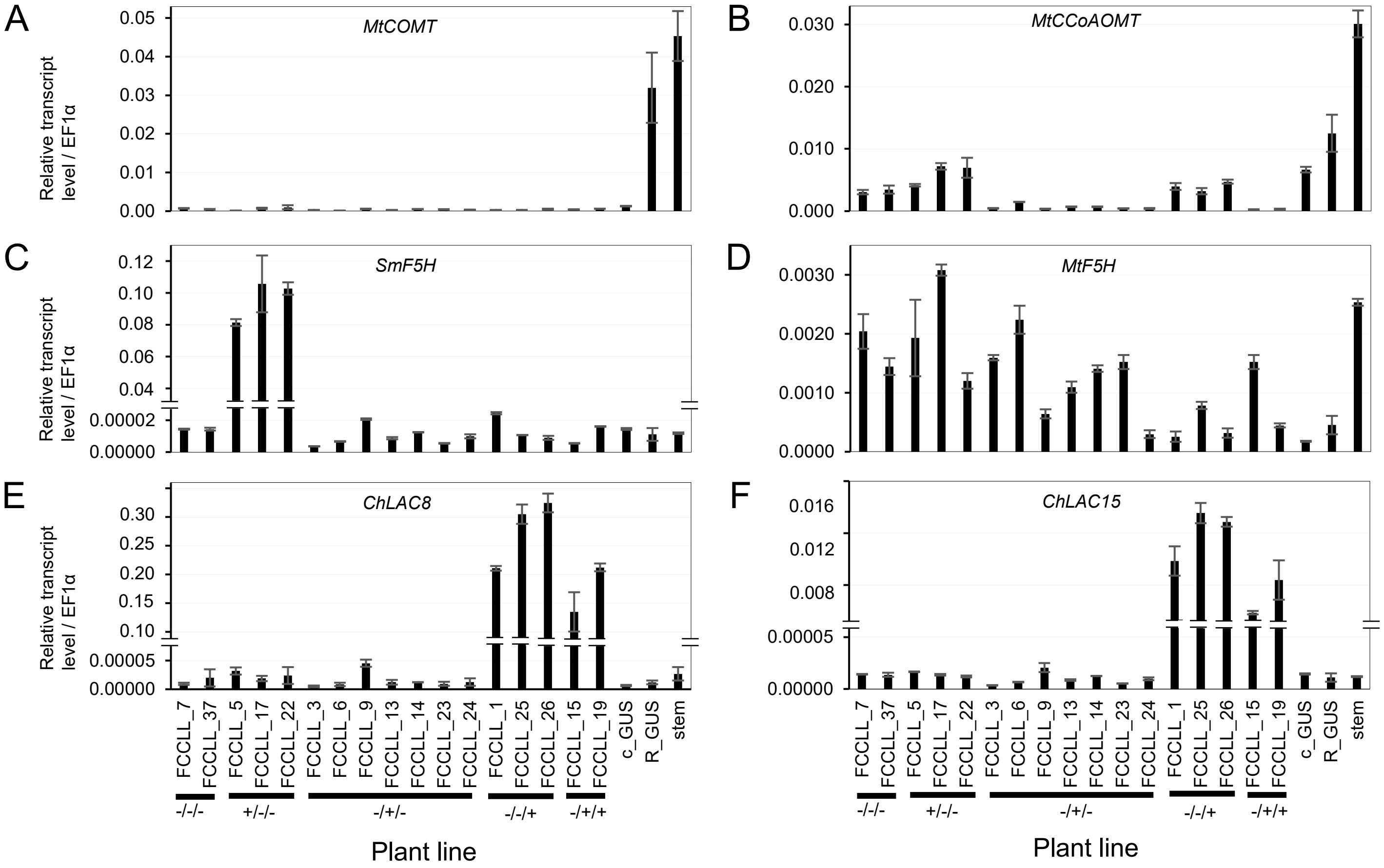

Supplemental Fig. S6. Monolignol pathway enzyme transcript levels in *M. truncatula* hairy roots engineered for expression of *SmF5H*-OX*, MtCOMT-MtCCoAOMT* RNAi and *ChLAC8-ChLAC15*-OX constructs in the *comt* mutant background.

Transcript levels were determined by qPCR analysis for *MtCOMT* (**A**), *MtCCoAOMT* (**B**), *SmF5H* (**C**), *MtF5H* (**D**), *ChLAC8* (**E**) and *ChLAC15* (**F**) transcripts. c_GUS, GUS control in *comt* mutant background; R_GUS, GUS control in R108 wild-type background; stem, stem tissue from R108 wild type. -/-/-, no transgenic event; +/-/-, transgenic roots only harboring SmF5H construct; -/+/-, transgenic roots only harboring *MtCOMT-MtCCoAOMT* RNAi construct; -/-/+, transgenic roots only harboring *ChLAC8-ChLAC15* overexpression construct; -/+/+, transgenic roots harboring both *MtCOMT-MtCCoAOMT* RNAi and *ChLAC8 -ChLAC15* overexpression constructs.
FCCLL, overexpression construct for *SmF5H* (F), RNAi construct for *MtCOMT-MtCCoAOMT* RNAi (CC) and overexpression construct for overexpression for *ChLAC8-ChLAC15* (LL). Data are means ± SD derived from three biological replicates.

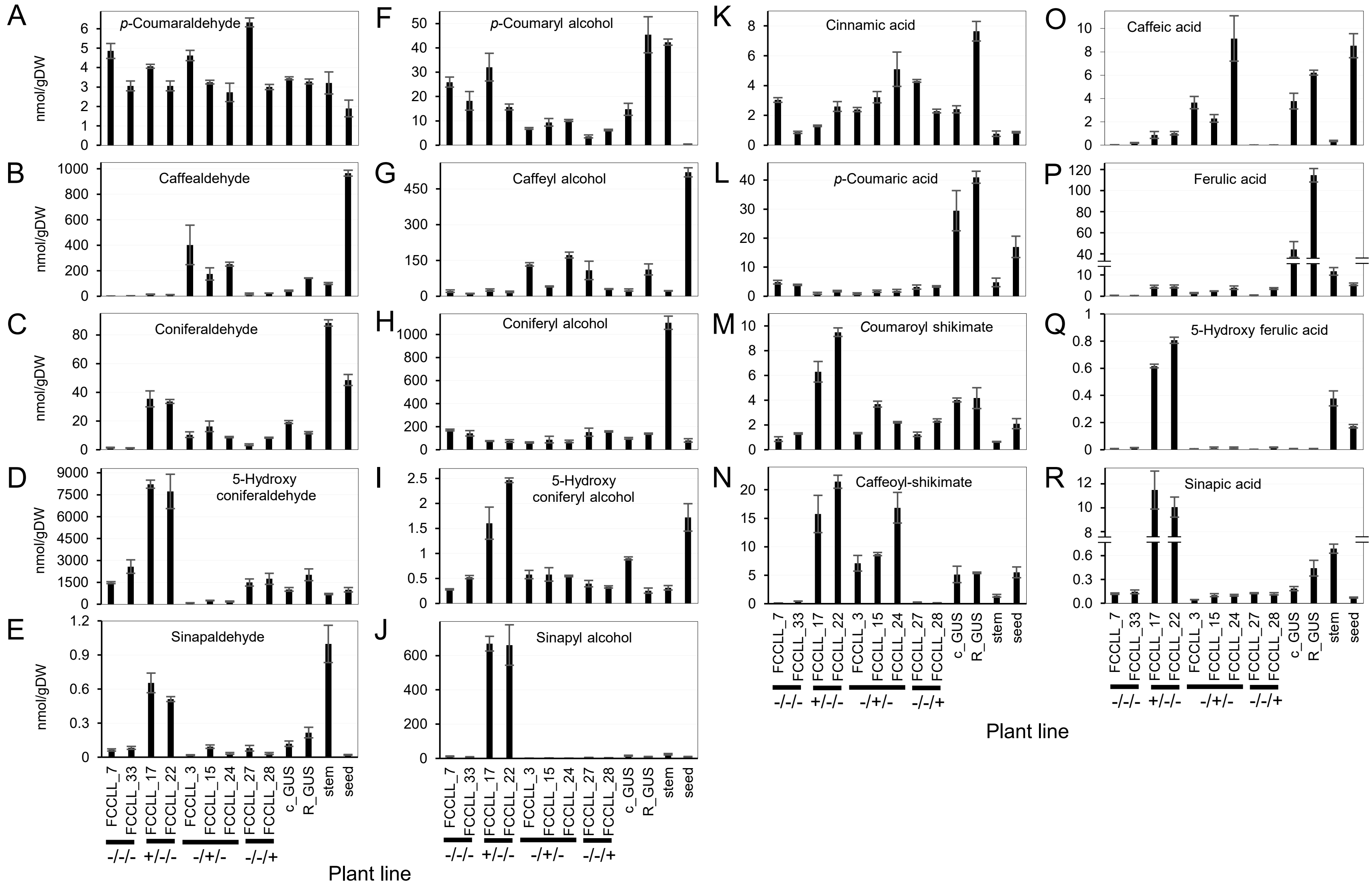

Supplemental Fig. S7. Levels of monolignol pathway intermediates in *M. truncatula* hairy roots engineered for *SmF5H* overexpression, *MtCOMT-MtCCoAOMT*RNAi and *ChLAC8-ChLAC15*overexpression in the *comt*mutant background. Metabolite levels were determined by LC-MS transitions pre-determined for individual compounds and quantified by comparison to authentic standards. Compounds are arranged in columns basedon mainly on chemical class. Structures are shown in Fig. 1. stem, wild-type R108 mature stem. c_GUS, GUS control in *comt*mutant background; R_GUS, GUS control in R108 wild-type background; seed, seeds of *Cleome hasserliana*(C-lignin control). -/-/-, +/-/-, -/+/-, -/-/+ represent genotyping results for the presence or absence of the SmF5H-OX/ MtCOMT-MtCCoAOMTRNAi/ ChLAC8-ChLAC15-OXconstructs as indicated in the legend to Fig. 9. FCCLL, overexpression construct for SmF5H (F), RNAi construct for MtCOMT-MtCCoAOMTRNAi (CC) and overexpression construct for overexpression for ChLAC8-ChLAC15 (LL). Data are means ±SD derived from three biological replicates.

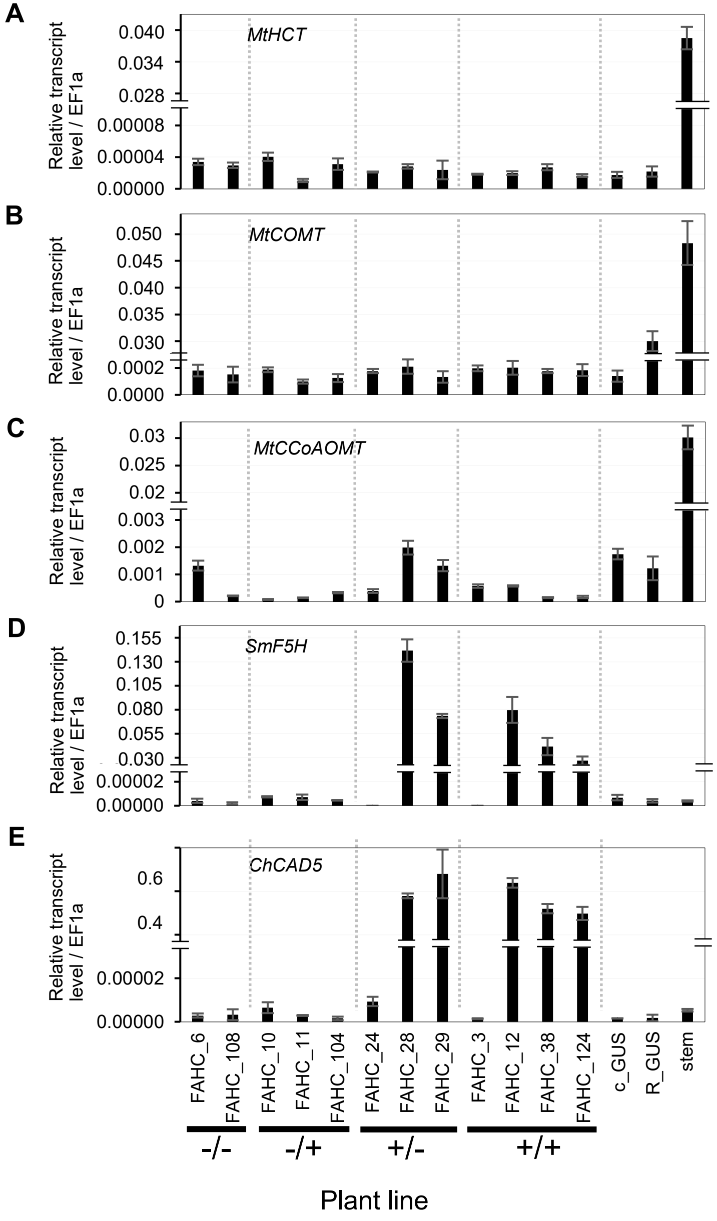

Supplemental Fig. S8. Monolignol pathway enzyme transcript levels in *M. truncatula* hairy roots engineered for expression of *SmF5H-ChCAD5* OX + *HCT-COMT* RNAi in the *comt* mutant background. Transcript levels were determined by qPCR analysis for *MtHCT* (**A**), *MtCOMT* (**B**), *MtCCoAOMT* (**C**), *SmF5H* (**D**) and *ChCAD5* (**E**) transcripts. c_GUS, GUS control in *comt* mutant background; R_GUS, GUS control in R108 wild-type background. FAHC, overexpression construct for *SmF5H* (F) and *ChCAD5* (A) and RNAi construct *for MtHCT* (H) and *MtCOMT* (C). Genotyping led to selection of plants with no transgenes -/-, non-transgenic event; -/+, transgenic roots with only the *MtHCT-MtCOMT* construct; +/-, transgenic roots with only the *SmF5H-ChCAD5* overexpression construct; +/+, transgenic roots with both *SmF5H-ChCAD5* overexpression and *MtHCT-MtCOMT* RNAi constructs. Data are means ± SD derived from three biological replicates.

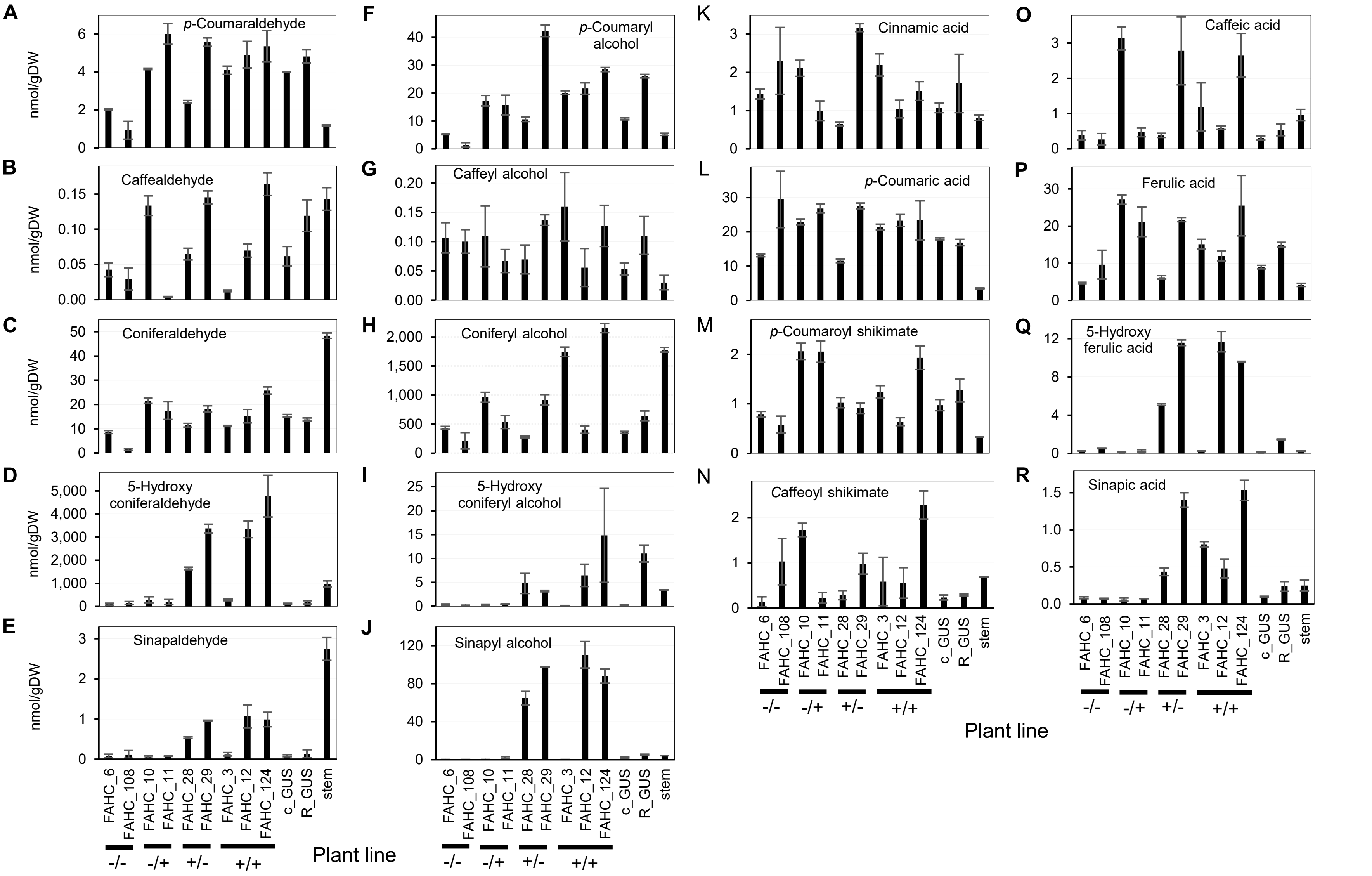

Supplemental Fig. S9. Monolignol pathway metabolite levels in selected *M. truncatula SmF5H-ChCAD5* overexpression / *MtHCT-MtCOMT* RNAi hairy roots. Metabolite levels were determined by LC-MS transitions pre-determined for individual compounds and quantified by comparison to authentic standards. Compounds are arranged in columns based on mainly on chemical class. Structures are shown in Fig. 1. stem, wild-type R108 mature stem. c_GUS, GUS control in *comt* mutant background; R_GUS, GUS control in R108 wild-type background. FAHC,transformed with overexpression construct for *SmF5H* (F) and *ChCAD5* (A) and RNAi construct for *MtHCT* (H) and *MtCOMT* (C). Genotyping led to selection of plants with no transgenes (-/-), RNAi but not OX transgene (-/+), OX but no RNAi transgene (+/-), and the presence of both transgenes (+/+). Transgene constructs are shown in Additional File 1: Fig. S2, and transcript levels of the targeted genes in each line shown in Additional File 1: Fig. S8. Data are means ± SD derived from three biological replicates.

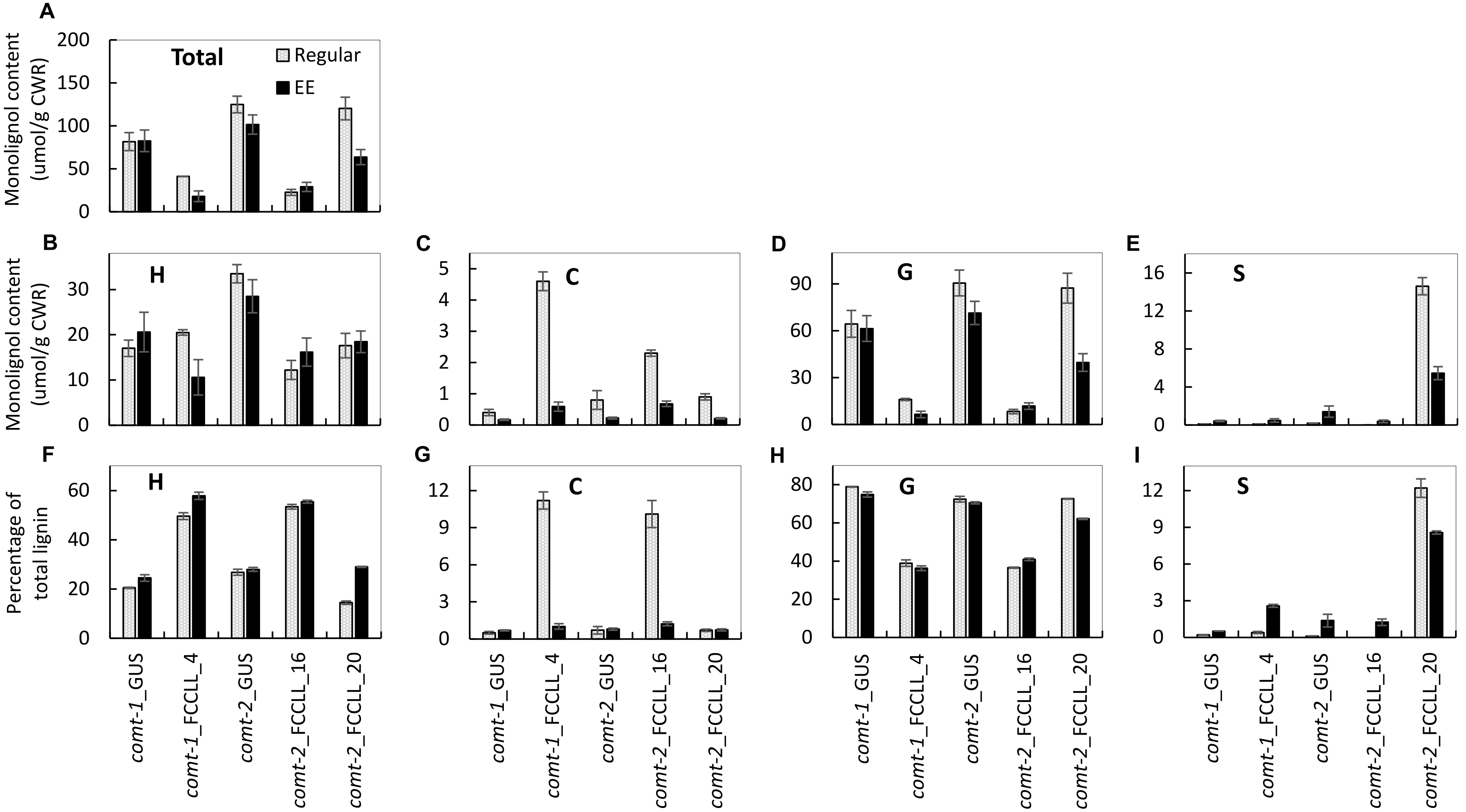

Supplemental Fig. S10. Differential extractability of C- and G-lignins from *M. truncatula* hairy roots as determined by thioacidolysis. (**A**) Total monomer yields from extractive-free, freeze-dried root samples from the 6 independent hairy root lines shown, before (Regular = regular sample preparation for thioacidolyis) and after (EE) enzymatic hydrolysis with cellulase and EDTA treatment. (**B**-**E**) Total monolignol thioacidolysis yields for H units (**B**), C units (**C**), G units (**D**) and S units **(E**) before and after EE. (**F**-**I**) Percentage composition (of total lignin) of H units (**F**), C units (**G**), G units (**H**) and S units (**I**) before and after EE. Hairy root lines were high C content (FCCLL_4 and FCCLL_16), high S content (FCCLL_20), and two GUS controls. FCCLL, overexpression construct for *SmF5H* (F), RNAi construct for *MtCOMT-MtCCoAOMT* RNAi (CC) and overexpression construct for overexpression for *ChLAC8-ChLAC15* (LL). Data are means ± SD derived from three biological replicates.

Supplemental Table S1. List of primers used in this study

<table>
<tr><th>Type</th><th>Gene</th><th>Primer Name</th><th>Sequence</th><th>Use</th></tr>
<tr><td rowspan="14">Gene cloning</td><td rowspan="2">ChLAC8</td><td>ChLAC8_F3</td><td>ATGGCCAGTTTTGAGTGCTTTCT</td><td rowspan="2">LAC8-OX</td></tr>
<tr><td>ChLAC8_R3</td><td>TTAATATTGGTCGTACGTAGGATG</td></tr>
<tr><td rowspan="6">ChLAC15</td><td>LAC15_F1</td><td>TCTAGTCGACCATATGGGAGAGCTCAAGCTTGCATGCCTGCA</td><td rowspan="6">LAC8-LAC15_OX</td></tr>
<tr><td>LAC15_R1a</td><td>GCTTCGCTGAGCAGTGACATGGCTATCGTTCGTAAATGGTGA</td></tr>
<tr><td>LAC15_F2a</td><td>TCACCATTTACGAACGATAGCCATGTCACTGCTCAGCGAAGC</td></tr>
<tr><td>LAC15_R2</td><td>TGCAGCCGGGCGGCGCTTAACAAGTTGGCATGTGAG</td></tr>
<tr><td>LAC15_F3</td><td>CTCACATGCCAACTTGTTAAGCGCCGCCCGGCTGCA</td></tr>
<tr><td>LAC15_R3a</td><td>GATCCAAGCTCAAGCTAAGCTTGCATGCCTGCAGGTC</td></tr>
<tr><td rowspan="6">ChCAD5</td><td>cleoCAD5_p35S_1F</td><td>AAGTTCATTTCATTTGGAGAGGACATGGGAAGGCATGAAGGAGAG</td><td rowspan="6">ChCAD5-SmF5H_OX</td></tr>
<tr><td>p35S_ pCC0916_1F</td><td>AAGCTAGCTTGCATGCCTGCAGTGAGACTTTTCAACAAAGGGTAATA</td></tr>
<tr><td>p35S_ cleoCAD5_1R</td><td>CTCTCCTTCATGCCTTCCCATGTCCTCTCCAAATGAAATGAACTT</td></tr>
<tr><td>cleoCAD5_NOS_1R1</td><td>TTTATTGCCAAATGTTTGAACGATCTCACACATTGCTTCCGGCGAC</td></tr>
<tr><td>NOS_ cleoCAD5_1F1</td><td>GTCGCCGGAAGCAATGTGTGAGATCGTTCAAACATTTGGCAATAAA</td></tr>
<tr><td>NOS_ pCC0916_1R</td><td>TTTCTCAGTTCCTCCTCTAAGCTGATCTAGTAACATAGATGACACC</td></tr>
<tr><td rowspan="18">qRT-PCR</td><td rowspan="2">MtHCT</td><td>MtHCT_QF3</td><td>ATGATCATAAACGTTAGAGATTCGA</td><td rowspan="2"></td></tr>
<tr><td>MtHCT_QR3</td><td>ATAAAAGTAAACACTTGGTGTATGG</td></tr>
<tr><td rowspan="2">MtCOMT</td><td>MtCOMT_QF3</td><td>ATGGGTTCAACAGGTGAAACTCA</td><td rowspan="2"></td></tr>
<tr><td>MtCOMT_QR3</td><td>TAAGAGATCAAGTTCAAGAGCTGA</td></tr>
<tr><td rowspan="2">MtCCoAOMT</td><td>MtCCoAOMT_Q3F</td><td>GGAAGTATGTTAGGTACTATAGAG</td><td rowspan="2"></td></tr>
<tr><td>MtCCoAOMT_Q3R</td><td>TTACTTGATCCGACGGCAGATAG</td></tr>
<tr><td rowspan="2">MtF5H</td><td>MtF5H_QF2</td><td>TTCCTCCACATGGTAGCTATTTC</td><td rowspan="2"></td></tr>
<tr><td>MtF5H_QR2</td><td>GCACGGTCGTAAGTTAGGTATT</td></tr>
<tr><td rowspan="2">MtCAD</td><td>MtCAD_QF1</td><td>CCATGATTGAAATTGTGACCATG</td><td rowspan="2"></td></tr>
<tr><td>MtCAD_QR1</td><td>CAAATTTACTTCCTTTGACATCC</td></tr>
<tr><td rowspan="2">SmF5H</td><td>SmF5H_Q2F</td><td>CATGCGAAAGCCACACATCC</td><td rowspan="2"></td></tr>
<tr><td>SmF5H_Q2R</td><td>GTGCCTCCTTGACGATACAC</td></tr>
<tr><td rowspan="2">ChCAD5</td><td>chCAD5_Q2F</td><td>CATCCGACAAGAAGAGACGAG</td><td rowspan="2"></td></tr>
<tr><td>chCAD5_Q2R</td><td>TGATGTAGTCGAGAGAATCCG</td></tr>
<tr><td rowspan="2">ChLAC8</td><td>ChLAC8_Q2F</td><td>AAGCTGAACTTGGTTAACCCGC</td><td rowspan="2"></td></tr>
<tr><td>ChLAC8_Q2R</td><td>ATGTGGTCCGTTTTGGACGATG</td></tr>
<tr><td rowspan="2">ChLAC15</td><td>ChLAC15_Q3F</td><td>TGTCAGATTCAAAGTCGATAATCC</td><td rowspan="2"></td></tr>
<tr><td>ChLAC15_Q3R</td><td>ACGTTTCGTTTGGGAGCTTTCC</td></tr>
<tr><td rowspan="2">Genotyping</td><td rowspan="2">ChCAD5</td><td>PCC0916_1F</td><td>CACACAGGAAACAGCTATGACC</td><td rowspan="2">MtHCT-MtCOMT_RNA</td></tr>
<tr><td>chCAD5_4R</td><td>ACCATTGGGTAAAGAGCCTTGC</td></tr>
</table>

	SmF5H	SmF5H_4F	TCAGCCACAAGACGATCAAAGG	i and ChCAD5-SmF5H_OX
		PCC0916_2R	ACGTTGTAAAACGACGGCCAGT	
		chCAD5_2F	CACCATGGGAAGGCATGAAGGAGAG	
		SmF5H_5R	ATGTGGTTTTGAAACCGTGCGG	
	MtHCT and MtCOMT	PB7GWI_1F	AAGTTGACCGTGCTTGTCTCGA	
		HCT-COMT (RNAi-1)_2R	TGACCAAGAACGAGGATGGTGT	
		HCT-COMT _RNAi_5F	ATGGTAAGGTCAAGTCCACGAG	
		HCT-COMT _RNAi(2) _5R	CCTTCAAAGCCTTTGTAGGTCTC	
	MtCOMT and MtCCoA OMT	MtCCoAOMT RNAi_1F	ACAGCAAAACACCCATGGAACAT	MtCOMT-MtCCoAOMT_RNAi, ChCAD5-SmF5H_OX and LAC8-LAC15_OX
		ter35S_R	TATGCTCAACACATGAGCGAAAC	
		Hyg_1F	CTTGACTCTAGGGAATTAATTCCT	
		Hyg_1R	GAGTCTCATATTCACTCTCAACT	
	ChLAC8	pK7-LAC (35S)_1F	TAACTACACTATAGAGGTGACTGC	
		pK7-LAC_1R	AAATCGTCGGAGTACACTCCG	
